# Supplementary material for: Molecular insights into the powerful mucus-based adhesion of limpets (Patella vulgata L.)
Source: Open Biol. 2020 Jun 17;10(6):200019. doi: 10.1098/rsob.200019 (PMC7333891; doi:10.1098/rsob.200019)
Supplement: Supplementary Materials [file rsob200019supp1.pdf]

Molecular insights into the powerful mucus-based adhesion of limpets (*Patella vulgata* L.)

Victor Kang[1*], Birgit Lengerer[2,3], Ruddy Wattiez[4], Patrick Flammang[2]
https://dx.doi.org/10.1098/rsob.20.0019

[1]Department of Zoology, University of Cambridge, Cambridge, UK
[2]Biology of Marine Organisms and Biomimetics Unit, Research Institute for Biosciences, University of Mons, Mons 7000, Belgium
[3]Institute of Zoology, University of Innsbruck, 6020 Innsbruck, Austria
[4] Laboratory of Proteomics and Microbiology, Research Institute for Biosciences, University of Mons, Mons 7000, Belgium

*Correspondence: kwk22@cam.ac.uk

Supplementary Materials

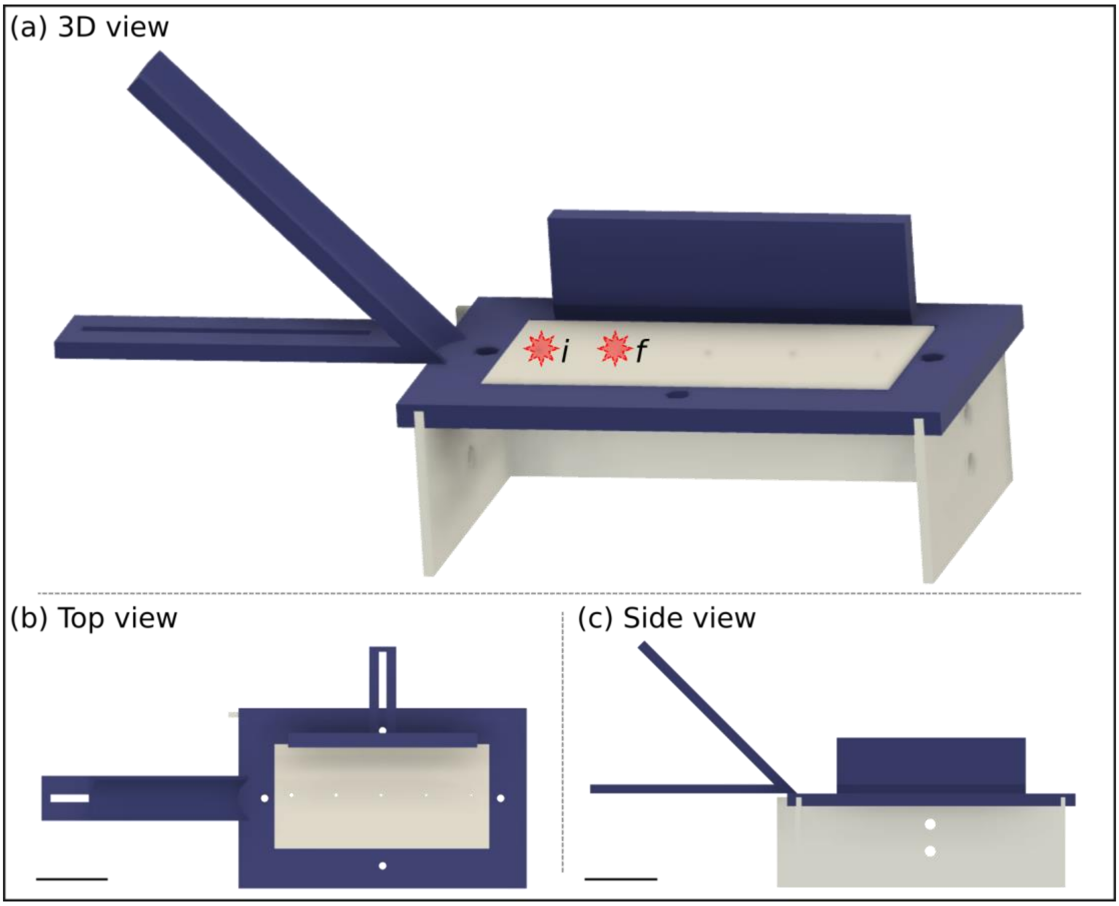

Supplementary Figure 1: Schematic of the *in vivo* pressure sensor rig made using 3D printed PLA components and laser-cut acrylic sheets. (a) 3D view, showing the 45° ramp used to accelerate the ball bearing. Red stars indicate initial *i* and final *f* position of limpets during free locomotion trials. (b) & (c) Top and side view of the set-up, respectively. Scale bar 40 mm.

Additional information on sequence quality check, trimming, and assembly
Quality check and trimming
Raw reads were filtered to remove reads with adaptors, reads with >5% unknown bases, and low quality reads (defined by BGI as reads in which the proportion of bases with a quality score < 10 was greater than 20%).

Assembly settings

The full software setting for read alignment using bowtie2, v2.2.5 was as follows: *Parameters: -q --phred64 --sensitive --dpad 0 --gbar 99999999 --mp 1,1 --np 1 --score-min L,0,-0.1 -I 1 -X 1000 --no-mixed --no-discordant -p 1 -k 200.*

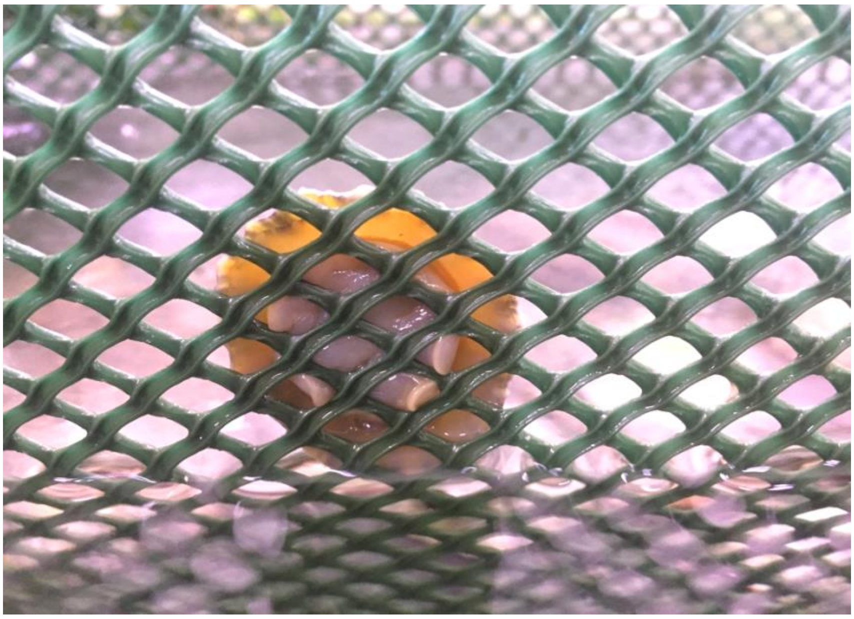

Supplementary Figure 2: Example of a limpet that managed to crawl up and adhere to plastic mesh.

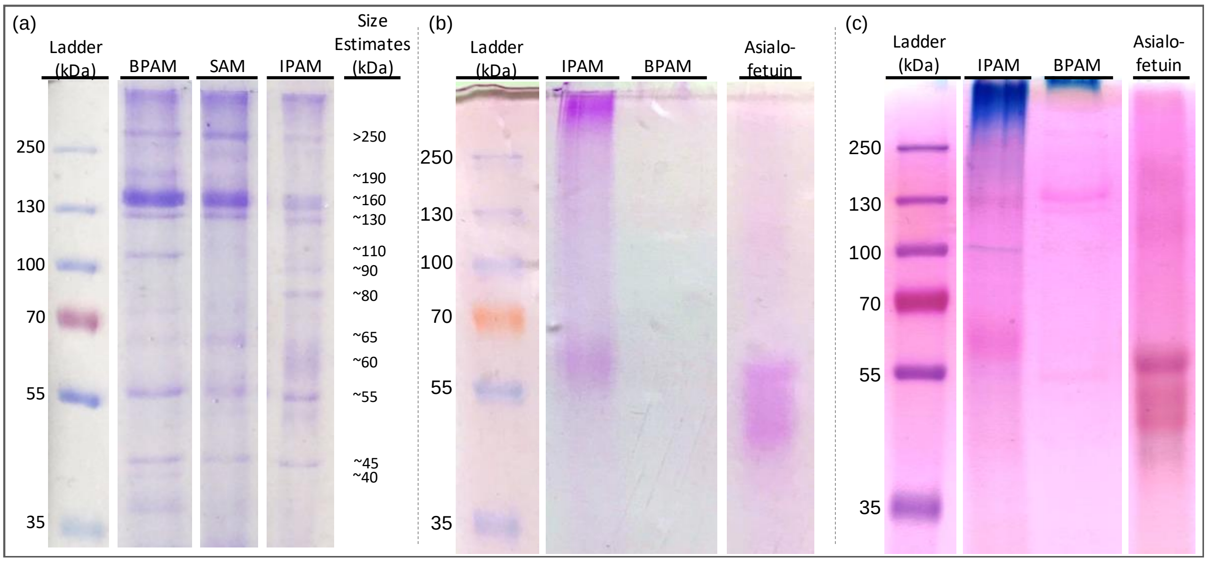

Supplementary Figure 3: SDS-PAGE gels of protein extracts from *P. vulgata* pedal sole mucus stained for additional information. BPAM: bulk primary adhesive mucus; SAM: secondary adhesive mucus; IPAM: interfacial primary adhesive mucus. (a) Coomassie Blue stain identified at least 11 prominent protein bands, ranging from ~40 to greater than 250 kDa. Note the approximate protein band sizes to the right of the gel image. (b) Smeared purple bands from PAS staining confirms that the presence of glycosylation for specific proteins within IPAM and not in BPAM. The strong smearing at the top of the gel indicates large complexes that failed to properly migrate into the gel. Asialo-fetuin from bovine serum used to as a reference glycoprotein to illustrate smearing pattern. (c) Multi-coloured bands from Stains-All highlight several differences between BPAM and IPAM (blue for highly acidic proteins and Ca^{2+}-binding proteins, purple for intact proteoglycans, and pink for weakly acidic proteins).

Supplementary Table 1: Transcriptome sequencing and assembly results of mRNA isolated from limpet pedal sole.

**Sequencing output**	
Total raw reads (M)	256.35
Total clean reads (M)	220.91
Total clean bases (Gbp)	33.14
High quality reads	
Q20 (%)	97.74%
Q30 (%)	94.30%

**De novo assembly result**	
Transcript number	86,396
Total size (bp)	81,369,009
Transcript N50 length (bp)	1,860
Transcript mean length (bp)	941
GC percentage (%)	35.59%

Supplementary Table 2: Functional annotation across seven databases.

Values	Total	Nr	Nt	SwissProt	KEGG	KOG	InterPro	GO	Intersection	Overall
**Number**	86,396	32,756	12,014	22,852	24,514	21,018	24,900	17,754	4,510	37,261
**Percentage**	100%	37.91%	13.91%	26.45%	28.37%	24.33%	28.82%	20.55%	5.22%	43.13%

Supplementary Table 3: List of limpet protein sequences tested with ISH.

Trinity assigned ID	Manuscript ID
Unigene47813	P-vulgata_1
Unigene46021	P-vulgata_2
CL3399.Contig1	P-vulgata_3
CL6799.Contig2	P-vulgata_4
CL6346.Contig2	P-vulgata_6
CL4559.Contig2	NA*
Unigene2524	NA
Unigene11734	P-vulgata_7
Unigene35700	NA
CL3409.Contig2	NA
Unigene36817	P-vulgata_8
CL489.Contig2	NA
CL5700.Contig1	P-vulgata_10
Unigene11944	P-vulgata_11
CL1231.Contig2	P-vulgata_12
Unigene15438	P-vulgata_14

*: Not applicable (NA), where a manuscript ID was not generated for a given Trinity-assigned protein ID as it was not included in downstream manual annotation and ISH was unsuccessful.

List of primers used to synthesise ISH probes

Manuscript ID	Primer Sequence
FW_P-vulgata_1_T7	GGATCCTAATACGACTCACTATAGGTTCCACATCAACCTTGTTCACC
FW_P-vulgata_2	TTTACGAATGTACGGCATCACC
FW_P-vulgata_3	TGTGATGGAGCAAACTGTACCC
FW_P-vulgata_4	GAAAACGACCATGTTCCAATCC
FW_P-vulgata_6_T7	GGATCCTAATACGACTCACTATAGGGCTGGGTTTCTCATTAGGTTCG
FW_P-vulgata_7_T7	GGATCCTAATACGACTCACTATAGGTCCTGGTGATTTCAATGGTAGC
FW_P-vulgata_8	ATTCTATCGGCGCATATTACGG
FW_P-vulgata_10	CAAAAAGAACGTGTTCCAGTCG
FW_P-vulgata_11	CTGTTGTGGTTTACAACCATGC
FW_P-vulgata_12_T7	GGATCCTAATACGACTCACTATAGGCACCCACCACAGTAGTTAGAGACG
FW_P-vulgata_14_T7	GGATCCTAATACGACTCACTATAGGTTCCACTATGCGACACAAGAGC
FW_PV2524_T7	GGATCCTAATACGACTCACTATAGGTTTCGATCTAGGCAGAGAAATCC
FW_PV3409_T7	GGATCCTAATACGACTCACTATAGGTTCGAATTTTGCTCAACAGTGC
FW_PV35700_T7	GGATCCTAATACGACTCACTATAGGGGCCTCAAAACAGGTACATTGG
FW_PV4559	TTGTCTTGGAAGGAAACACAGC
FW_PV489	GGAGATATTGGTGCTGTTGACG
RV_P-vulgata_1	CAAAAGAAAAGGACGCAGATCC
RV_P-vulgata_2_T7	GGATCCTAATACGACTCACTATAGGCAATGCCACAGTTACAGTTCC
RV_P-vulgata_3_T7	GGATCCTAATACGACTCACTATAGGTGAAATCTTTTCCAGGGTTTGG
RV_P-vulgata_4_T7	GGATCCTAATACGACTCACTATAGGCAGGGTTTCCATCGAAGTTACC
RV_P-vulgata_6	TGCAAATGTGATATGCTCAACG
RV_P-vulgata_7	GATACGTGGTCCCAGAGAATCC
RV_P-vulgata_8_T7	GGATCCTAATACGACTCACTATAGGAAAGGTACAATGTGTGCGATCC
RV_P-vulgata_10_T7	GGATCCTAATACGACTCACTATAGGTAAACGTGTCTTGGATGGATGG
RV_P-vulgata_11_T7	GGATCCTAATACGACTCACTATAGGACTGGCCGTACTTGTCAAAAGG
RV_P-vulgata_12	AAATTACGTCGGAATGAATTAGCC
RV_P-vulgata_14	CCTTACGAAAAGGATGGATTCG
RV_PV2524	ACTAGCTCTGGTGGCTTACACG
RV_PV3409	GTAGAATGTACCGGCGAAGAGC
RV_PV35700	TACATCGAGTTGTCCCAACAGC
RV_PV4559_T7	GGATCCTAATACGACTCACTATAGGTTTCCAGGTCACCATCATAACG
RV_PV789_T7	GGATCCTAATACGACTCACTATAGGTCAATTTTATTCGGACGTGATCC

Supplementary Figure 4: Clustal alignments for SCO-spondin, Reprolysin, and Tc-MUC.

(a) Multiple SCO-spondin precursors and *P-vulgata_3* alignment

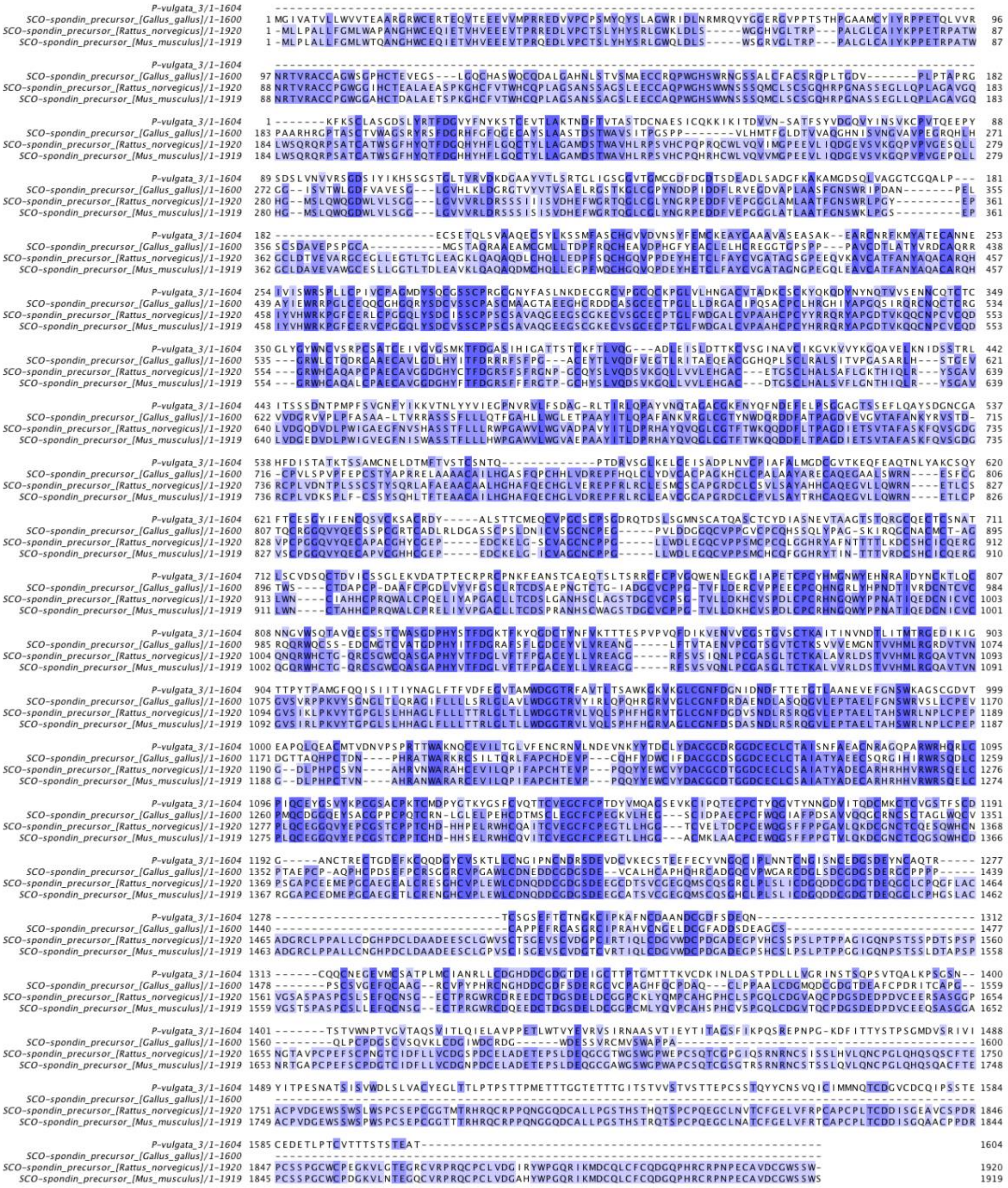

SCO-spondin *Gallus gallus* (truncated) and *P-vulgata_3* alignment showing SCOR regions

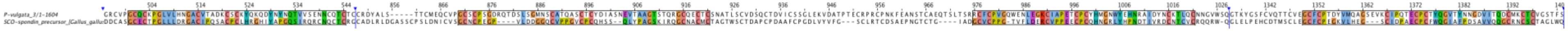

(b) Reprolysin CDD conserved sequences and *P-vulgata_8* alignment

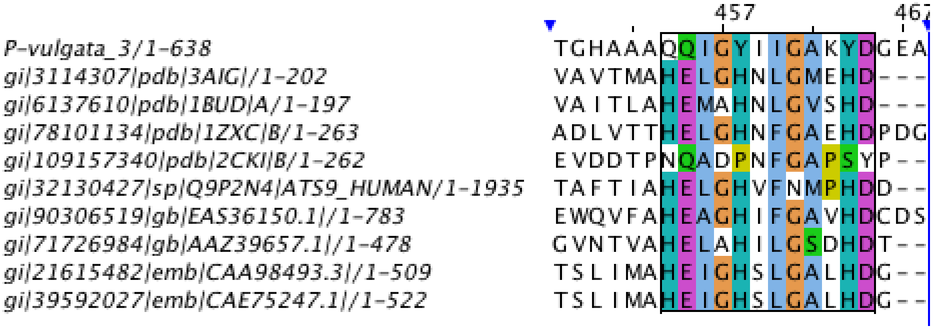

(c) Tc-MUC and *P-vulgata_14* alignment

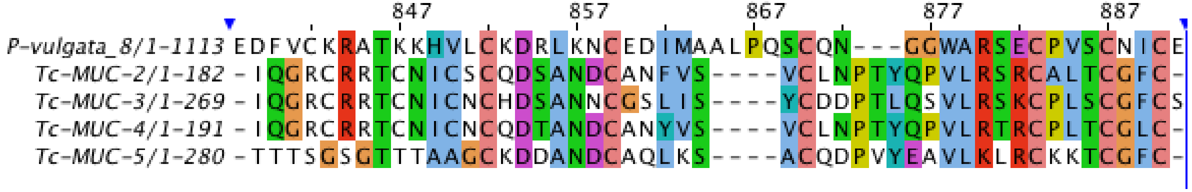

Detailed protocol of *in situ* hybridization (ISH)

RNA probe synthesis. Primers were designed with Primer3 (http://primer3.ut.ee, v4.1.0) and for antisense/sense probe production a T7/SP6 promoter region was added at the 5`end of the reverse/forward primers. cDNA was generated from isolated total RNA of the limpet tissue, (same limpet individual as the one sent for transcriptome sequencing) using Transcriptor First Strand cDNA Synthesis Kit (Roche). PCR reactions were performed with Q5® High-Fidelity DNA Polymerase (NEB) and their correct length verified on a 1 % agarose gel. The PCR products were purified with Wizard® SV Gel and PCR Clean-Up System (Promega). To synthesize single stranded digoxigenin-labelled RNA probes, T7/Sp6 polymerase (Promega) and DIG labelling mix (Roche) were used according to manufactures' protocols. DIG-labelled RNA probes were precipitated with isopropanol and bands length and intensity checked on a 1 % agarose gel. RNA probes were diluted in hybmix to an estimated concentration of 5ng/µl (based on gel images) and stored at -80°C until usage. Probes were used at a final concentration of approximately 0.2 ng/µl. We repeatedly observed unspecific background staining using sense probes, which should not be able to bind to any mRNA specifically (supplementary figure 4a). After extensive troubleshooting we realised that the precipitation with isopropanol was not sufficient to eliminate unbound DIG-labelled nucleotides, which were the source of this background staining. Please also see *background staining in the in situ hybridization experiments* addressing this issue.

Paraffin section *in situ* hybridization. For section *in situ* hybridization, Patella tissue was dissected and fixed in 4% PFA in PBS overnight. After several washes in PBS, the tissue was dehydrated in an ethanol series until 100% ethanol. Tissue pieces were treated with xylene for 8 and 5 min and immediately placed in 60°C preheated liquid paraffin wax. The tissue was moved to fresh preheated paraffin wax every hour for three times and left at 60°C overnight. Afterwards, the samples were moved to room temperature for the paraffin blocks to harden. The paraffin-embedded tissue was then cut into 14 µm thick sections using a Microm HM 340 E microtome. The sections were placed on Superfrost ultra plus® slides (Thermo scientific) and heated in an oven at 60 °C for 1 h and allowed to cool down for 15 min. After dewaxing in xylene and rehydration, sections were postfixed in 4% PFA in PBS for 20 min, washed several times in PBS and treated with 0.2 M HCl for 10 min. Following washes in PBS, sections were treated with Proteinase K (20 µg/ml) for 20 min. Sections were postfixed in 4 % PFA in PBS for 5 min, washed in PBS and placed in 0.1 M triethanolamine (pH 8) containing 0.5 % acetic anhydride for 20 min (exchanging the solution after 10 min). Afterwards sections were washed and gradually dehydrated with ethanol. To dry the sections, Chloroform was dropped on the sections and allowed to evaporate. Slides were placed in a humidity chamber and incubated with hybmix at 55 °C for 30 min. Sections were incubated with RNA probes diluted in hybmix (0.2 ng/µl) overnight at 55 °C. To avoid evaporation of the liquid, sections were covered with fresh parafilm pieces during this step. The following day the sections were incubated in 50 % formamide in 2x SSC at 62 °C (3 x 20 min). After washes with 1x SSC and PBS, sections were blocked in blocking reagent (Roche) for 1 h at 4 °C. Sections were incubated with Anti-digoxigenin-AP Fab fragments (Roche) 1:2000 in blocking reagent overnight at 4 °C. Following several washes, the signal was developed using the NBT/BCIP system (Roche) at 37 °C. Colour reaction was stopped with ethanol and sections washed several times with PBS. Sections were mounted in Mowiol® 4–88 (Roth, Germany), prepared according to the manufacturer's protocol and images were taken with a Zeiss Axioscope A1 microscope.

Background staining in the *in situ* hybridization experiments. For ISH negative control samples, we used DIG-labelled RNA sense probes that should not bind to any mRNA sequences. However, we repeatedly observed unspecific background staining in the negative controls in glands ~100-150 µm from the epithelium, in the epithelium and secreted mucus (supplementary figure 4a). No staining was observed when no ISH probes were added to the samples (not shown). For interpretation of ISH results in this study, we only considered probes that consistently stained differently to the background in two to three repeated ISH experiments. Using *P-vulgata_3* as an example (supplementary figure 4c; modified version of Figure 3b), the positive stainings were distinct in intensity and locality compared to background stainings (supplementary figure 4a). Subsequent troubleshooting by B.L. in Mons, where all the ISH experiments were conducted, identified the probable cause as short DIG-labelled nucleotides that freely bound to unspecific regions of the tissue. When negative control probes were additionally purified through a Micro Bio-Spin® Chromatography Column (Biorad) and re-tested, the unspecific background staining issue was

resolved (supplementary figure 4b). Unfortunately, data collection for the project had concluded several months prior to this clarification, and the lead author had already returned to Cambridge, UK, hence ISH could not be repeated again. Nevertheless, by carefully considering the differences between background and signal staining patterns and by upholding a high threshold for inclusion, we are confident that we have reported valid results for the five candidate probes (shown in Figure 3 of the main text).

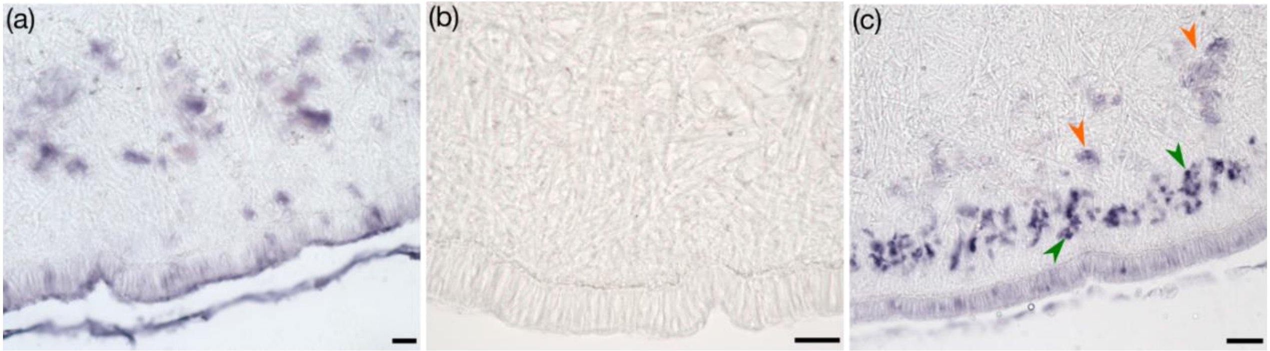

Supplementary Figure 5: Unspecific background staining due to DIG-labelled nucleotides present in the ISH probes. (a) Unspecific background stains in the negative control samples were diffuse and localised to ~100-150 µm from the epithelium. Scale bar 20 µm. (b) When negative control probes were additionally purified through microspin columns, background stains were eradicated. Scale bar 20 µm. (c) *P-vulgata_3* staining, showing the distinct staining pattern between the signal (green arrows) and the background (orange). Based on our prior experiences with ISH, the sharp intense stains indicated by the green arrows are indicative of positive stains. Additionally, the background and positive stains were segregated by location within the tissue. ISH probes that did not fit these criteria were not included in the analysis.

Additional information on PX26-30DV pressure sensor calibration: The pressure sensor was calibrated by connecting a syringe pump to an analogue pressure gauge (WIKA Instruments Ltd, UK), which in turn was connected to the sensor port. The syringe pump was used to increase the pressure (relative to ambient) from 0 to +40 kPa in 10 kPa bar steps, and the same to decrease the pressure from 0 to -40 kPa. Sensor output voltage was then plotted against pressure, and a linear regression (intercept set to 0) was fit to obtain the relationship Pressure (kPa) = 8.6979 * Volts (V), with a Pearson's correlation $r^2 = 0.9995$ (see below). This linear model was used to analyse the in vivo sub-pedal pressures from *P. vulgata*.

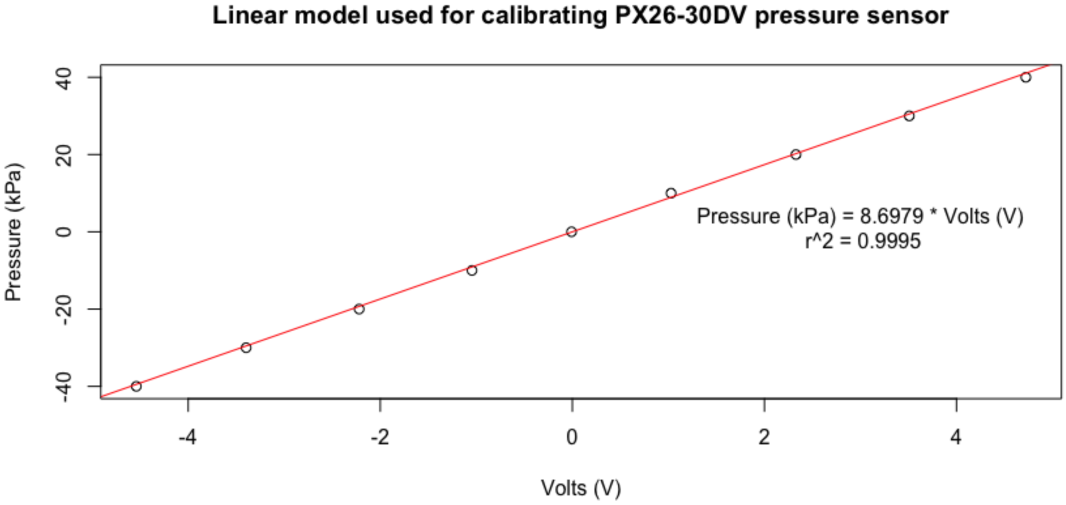

Additional information for manual vertical pull-off experiments: for the trial shown in Figure 3c, the limpet had been disturbed previously and had clamped down, corresponding to the slight negative pressure (around -1.4 kPa). The pull-off force was applied by gripping the limpet's shell with fingers and pulling upwards starting from ~20s until detachment at ~25s. Because of this method, we cannot state with confidence whether the applied detachment force was constant during the whole duration. For this particular trial, we observed an initial negative peak of around -2 kPa, followed by a second much larger peak of -5.7 kPa, and then the pressure returned to -2 kPa for ~3 s before full detachment. One possible explanation for this second dip is that as the limpet was being pulled, it attempted to clamp down, which would result in a decrease in sub-pedal pressure. Once it relaxed, the pressure returned to around -1.4 kPa, before being fully detached. We wrote in the Discussion that clamping can lead to a sub-pedal pressure reduction, although this still requires adhesion between the contact area and the surface, which we propose is provided by adhesive mucus.

Encoding cDNA sequences of the fourteen annotated protein (see Supplementary Table 3 for identifiers)

>Unigene47813_L1_2_1
GAGTTTGCTTTAAGTATTTATTTATTTATAGTCCATAATTGAGACTAATTTATAGGATTTAAATATTCTGTTTGTTCATAATATATTATTGCATTTAAAATATAC
AGAAAGATTAGAAATTCTCAGCAGGAACATCTTTACACTTCTTGGTCTTTATCACCACAGACACAACTAAGTACTGACATAACCTCCTTTGGTATTTGTTGGG
ACAAACCATTGCCTGTACATGTAACATTAAACCTTATATATTCTACTTTTTCAGGTCGGCAACAAACATTAGTACCACCATCACAGTAGCCTTCATTACATAG
GGCTATAGTCATAACTTCAGAATTACATTCAGATTGTTGACTGCTACGAGAATATTTGTATGTTTGTTGGAATGGACCTTTTGAGCATGGTAAAGAAGATGG
TCGTTTTCCACCAGCTTGGCAAGCAGCTGGGTAGTATTCATTAGGATGGTAACTGACTTCTGAATTCTGGCACAAGGCTGTTCGTTCGAGATGACATCTGGA
AGAGTATGTTTTAGGTACGTCATCCACCTTTAAGACTCGTCCACAGTAAGGATCAGCAACTTTAGGACAATCACTAAAATCACATTCATTGGTTACAGGAGG
CATGACAGTGTCAGCAGAACAGCGTGGTAAATCACAGCTATCCATTTCACGTGTCGATTCACTTGTTAATAATTCTGGGTTTGGTCCAGTGTAGGTCCTAGT
TCTTTCTCTGACACCCATAGCGGTACCACAGTCTAAACCAATAGGTTGTGGTTTACAGTCTGACCAGATGGACCATGGCTCAAAATCAGATTCAGCAGGTGT
AGGTGCTTGCGTAGTTGTAGTGGTTGTCGGTGGATTGCATACACCTAAGGTATATATTGGTAATTCTTCAGGATTTTCTCTTTTCTGCAAACATATATGCATT
CTCAAATCACAGAGACTTGTGAAGTCTCTACCTCCTACACAGCATCCGCCTGTGGATAGTTCATAGTTACTACAGATATCATTTGAACTGCATGGATTTTTCG
TTGTGGAGGGAGCTTCTGTGGTTGTGGTTGTAGTTGTACTTGTAGAGGTTGTTGGTTCGTTAGAGGGACATTTTCCATAATTAGCAATAGTAACGTTAATAT
TGGATTCACCATATTTGGCACAATTGACTTGATAAAGTTCTTTGTAATTGCTGTATGTTACACCATCAGAACCACATACCTGAGCAGGTGATCCATCTGTTGG
TGGTCTACATAGAGCTGTGTTACAAATACAACAATCGCTACCTATGGTAATTCCAGTATTACGTTGACATTTAAACCGGAATGGACCACCAGGACGGGCCA
TAGTGAAATCATAACCATAACAGCAAGAGCAAGCATAGGCTCCTGGTACATCTCGACATATGGAGTTTTCTGGGCATCTCGGTGTTTCTGATTCTTCCTGAC
ATTCATTGATATTGGCACAAACATTGAGTGAATTTTTCCTGTATCCGCTCTTACACTCGCATTCAAAAGAACCCGGTGTATTTTTACATTGCTCATTGATTCTA
CAGTCGTGTAGATTAGCCTGGCATTCATCCAAATCAACACATGTGTCACAATCAGAGACATAACCGGTTTTACATAGACAGACCTTCACATCATCCTCATCC
AGTTGTACAGTAGCACAGAAACTGCTGTCTGTTTCATCACCTTTCTTGCATTCAACCTTAGCATCACCTAGGTATCCATCAATACACACAACAGCTATTTCATT
GTTTTTAGTACCACATTTGGAATTTGGTATAAGTGTATTTTCACGTTCTTCAACAACGCCTGCTTTACATACAAACAATTTAGAACAGTCAGCATTTAACCATT
CGTCATTAATCTCAAAATATCGTTCTGAATTTGGATCCTCACATCCACATTCAGAGAGAGGTATACATCCTCCATTTTGCAGGAAGAATCCAGCACGACATAT
ACATTGTGGGTTACGAGGATTTGCTTCACCTTCATTAGCTGCATTAGGATATTTGTTACTACATGTAGGGAGTTCCTGGGGTGGTTTAAAACTGAATACCAT
ATTAGGTCCACAGTCTTCAACACAACCAGTTCGCATCAAAGATTCAGCTCTATCTACTTGAGACAATATGTAATCTTTACAGTCTACAGAGATTGTCTCAGTT
AACACGTCACAGAAATCCACCCCCTCATTGCAGGTCTTTGTAATACATAACTCATATAATCCTTGTGTATTTGAACAGTTATTAAATGGACCGTCAACAATTA
TACCACATTTGTTAGCAGCAGACATTGTAGAAGGGCATGCGCCTGGTGGAAGAATTTCTGGAGGGGGTGTTTCACCAGGGCTAGGATTAGGAACTCCAGT
TGTTGGGATAGATGGTGGAGCATCTGGTACGGTTGTTGGACTAGGTTGATTAACTGGCTGTGATGGTTGGGTTTCTGGATTTGGGGGAGTTGCTGGTCCA
GGACCTGTGGGTGACTCCGAATTTGGTGGTGGTGGTGTTAATTCTGGTGGTGGTGTTGGTGGTTCTTTATCAGTATTTTCTGTATTTGACTCTCCAGAGCCA
AGTTCACTACATCTATCAAATGCTTGAGAAACGTCCATACTGTCTAGCAAATCACAGGATCCACAAAAACCAACAACAGATGTTATCTCTGTTTCCAAATTAA
CTGTATCTACCATAACATTGAGTTTGCCATCATTGCTAGAAACTAATAATTCTCTAGCTGACTGCATAATCTTAATATCGGTGTACTTTCTTCCATCACTGTAG
AATTCCCCATTAACCTCAACTCCAAGTGTTGTTACTGAAACAACTGTGTCTTGAACCTTTATTCTAATTGTTACGACATATGTGCCTCCACTTTTAGCTTTAAT
AACCTTTACATTTAATTTTTCCGGAGAAACACAGTCTTCTACTAAGGTAACTGTACAATCTCCAACTTCTTGCGATGGTAAATCGAGCGAATTAAATCTGGTA
ATCATGAAAGTATTACCATCACTGGGCATACTGAATTGACATTTTTCTGTGGCTAAACATGTTTCATCGTGAAGAATGTCGTCTGGATCTGAGCAATGACAG
CTGAAACTGCCCCAATCATCTGTACATTCAGTTCCATCTGGACACAAATCAACACCCACTTCGCTGCATTCATCGATATTTTTTTCACATTTTCCTTGGACAGT
ATAATCTGTACATGAGTTCATATCACCACCATAACAAGTATAGAGTTCCCCGCTTGGTCGAGCTTTACATACTGTTTCTGTAACAGAGGTTGCATCGGAAAC
AGGATCACATTTTACATCCATAGGGAACGTACAGATACAGGTAGGATCATCAGTTCGGATTTCTTGGATGCTTTCAACTTCATTTGCATCAGTTACATCACTT
CCATCACAATTACCAGTGTCTACAGTTTTAATTTCATCTGGTTTCTGTTCACAGATGTACTTCTTCTTTATTGTGCATTGTGTTAATTTTGGGGCTTCCGATTTT
GATAATCCAACGCATTTAAAACCAGTTGTTTCAGCACTGCTGTCTACCAGATTTGATGGGAGACGGAAATTCTCATATCCAGGATCTGTAAACTTATCATTG
CTTTCTTTTCTATTTGTGTTTACTCCAGCAACATAGAATTGACCTCCAAATCTCAATCGTCTCAATTTTTGAAATTCTGCTATTTCTGCTTCGCTTGTCAATTGA
AGCAAGTATCCTCCTCGTTGGTTACAGTCCTGTACAGCTGTACGCCATATCACCTTTTCCCTTGAGACATAAACACACTTATTACCAAATTTCTGGAATTCTCC
TGTACATGTGCTGATTTCTTCACACATATTTATCTCTACATTGAGTTTGAAGTTGTCCATACACGAAATTTCGGGTCTAGAATTATCTAGGTTACATACTCTTG
CAGTATCTGCAGGCATTGTAGCTTCCGTGACCAATTCACCATTAGATTTACATTTGACTTTCTCAGAACAGTTTGTATTCAATGTTACTTCATCAACATTTATC
ATAAATTTTTTCATGCCTGTTTCCGTTATCATTTCACAACCACATTCTTCTTTTTTGACACATTTAGTTCCTTTCCTGTAGTAGTCCCCTTCACATACACACATGT
CTCTGGGAGTACCACTACATGTTGTCTGTACGTTATGCATGTCTCCACATCTCTTATCACATTTAGCTAGATACATGACTTGCTCATTGGGTTCATCGCAATCC
AATTTACAGAAATCATCAGTTCGCCATTCCTTGGTTTCACCGGTTTTCAAATACAATTGTATAGATAAATCTACCATCTTTGAGCAAACAATTTTTTTTCGTTC
TTCATCATTCCCACTTTCTTCTTGACAGACATCAAATACACAATTGTCCACGAGCTCTGATATTTTCAAATCTTTAATTTTTTCTTCATATTCTGATACATATTCA
TTGTACAGAACTTTTAGTTGACCACAGAAATCTTCACTACCAAGTTTTTCTTCTGTCTCGGGACTACAATCTGTTACAGTTGGTACTGATTTACTACATCCAGC
ATTTTCATCATCATCGACTTGCCAATCATTGCCATATTCACTGGCAGAGGATAGCTCCCATCGTGATTTTCTATTCTGTCTGTATGTTGGGGTGAAAAAAGAG
CCAGCCCTCAATTTATATGCCTGTTCTTTTGATACACCAATATTTCCACAGATACCACACATGAGGCCGTCGTAGCCACTCGGTGTATCAATATATATCTTAG
ATCGGCCGTCAAACATGACCGACAAAGCATATGGAGTTGTAACTCTAGTTTCTTTACCACTGACTTCTGCTACGAAATATTTTGTCTCCTTTTTGTTTTCCACA
TCAACCTTGTTCACCTGTAGTTTTCCATTTCGAGCTAGTCTGATTGTGTCAAACGTAGAATCAAAGTTGTCCTTCTCATGGTAGATTGTAACATCACAATAAT
CGGTGCATGTGACATCTTTTCCCGGTTTGCCACACTTTCTATTCTTACATTCTACTTTAAACAGATCTAAATTCTTCAATTTGTCATTGTTTGTTTTGCAGACAC
CGGAGATGAGGTATTTGCATTTTCCGAAGAAATCCAGCATGTCCCCAGCATATGTTCTGCAATGAGGATCACCAGTACAGGAGCACAGATTTCCACTGACA
CATTGCTTAAGCTTGGAATTCCAATCTGCTCCAACTGGACATTTACAGAGGTAATGACCAAAAGTGTCTACACATTTCTGGCCACCGGGACAAGCATTAGAT

CCTGCTACATCGCATTCATTGACATCCTCAGGACATTCGAATTTCACCTTATAGTTCTTACAGGTTGGACCCTTGCAAATAAATTTATTTTTGGTAACTTCTAT
GCGATCTCCATCTCTTTCCTGTGGCTCATAGGTTATTTTAAATGGTTTCAAACACACAGGTTTCAATCTGATGATGGCCTGTTTAACAGTTAATTCGTATTTGT
TTCTTCCACGCTTTATAGGTTTACCTCCATCAACCAATACCTTCTTTCGCTTCTGAACTTCAAGAGGATCATATTCACACAATGGATATTTCAAATGATGGCAA
TGTGCGTTGATCTTAACACTGAATTTACCTGGAAGACGATTGCCAAATCCTGCAGTTGCCCTTGATCCCCTTGACCTATTTCTGGATCTGCGTCCTTTTCTTTT
GTTTCTCTTTGATCTGCCTGCCTTGTTTCGG
>Unigene46021_L1_2_1
GTTTAAGTTTTGAATATACAAAGTGACTAATAAGCTTGTAGTGAAGGCTTATCTTTGCCTTAATCTGTTCAGTTGATCAGACTGGGGTGAGCTAAGTTGCCG
ATAGTTCTGTAGGCAGTAGAAAAAAGGCGTTCCGACGCCAAGAAATCAAACAATATGATATTTATTGCTGCCGCGCTGATCTTTATTGCACCAATCGCTTCT
GCAGTCGAGATACCGTTACTCCCTGGTAATTGTTCCGAGGCGTATTTTTTCTGTCATGAATTAGCGAAATGCGAAGAGGTAAATGATACCAGTTTTAGATGT
GTGTGTGAAGCTGGAACAATTGGTGATGGATTAAAAGCAGAGGGTCAATCCGGCTGTAAACACACTGTGTTTCCATGTACTAATGAAGCTAACTGCCACAT
TGATGGATACTGTAATAAGACAGATTCTGCCCTTCCTGTGTGTAATTGCAAAAAAGGTTTTACGGGAGATGGCAAGAACAACTGTGATGATATCGACGAGT
GTTTAGCCAACCCATGTGACCAGAACGCCAACTGTCAAAACAACAAACCTGGTTTTAAATGTGTTTGTCTTCCAATGTTTACCGGTGATGGCTTCAAATGTT
CTCGAACATGTAGTGTAAAACAAGACTGTTTTCAACCTAATGGTGATTGTATAGAAAATTACTGTAAATGTAACGCTGGTTACACTGGTGATGCTTTTACTG
CTTGTGATGATATTGATGAATGTAAAACTGGAGACTTTGTCTGTACAGACCCAGCAGTTTGTGTTAATGAGCCCGGTACTTATACATGTGGCTGTCCAAATG
GTTACAAAGGAAATGGTGTAACATGTACAAAATTACCAAAAAACTGTCATGAAATATACCAAGAAGATCCTTCAGCAGTTAGTGGGAGAATGTTTGAAATA
GACCCTGATGGTACTGATCCGCTGGATGCCATGCAGGTGAAGTGTGAATTCAAAGATAATATTGGGATAACAATAGTTATAGCTTCAAAAAATACACCTCA
ACCAATTCACTCACAAGAAACACCAGTTGAATATAAGGGTATCTCACCAGAACAAATTAAAGGTCTTGTAGATAATTCAGAATTCTGTTATCAAACTCAATG
GATAGAATGTACTGGAGGATATAATCTATATCAAGACAATATGATGGAATGGACAGACTCTAATGGTACTACATATAGATCTTGGGGAGGATCTGGTCAG
GATGGAAATTGTGCTTGTGGTGTTCTTGGATTCTGCGATAAGGGCAAAACCTGTAATTGTGATGGTTCATCTACTGACACCGTGATTGATTTTGGAAAAATT
ATCGATTCGTCAAGATTACCAATCAGTTCCGTCAAATTCAAAGCTGGTTCGGGATCAAAGACAGCTAGCTATGTCATATCCGATGTAAAATGTGCACCCAAA
CCAATAGATTTATTACGAGATTGCGATGAAATAAAGAAAGATGGCGTGAGTGATAGTGGGCCACAAGTTATAGATATAGATGGTCCTGATGGTCCTCTCCA
ACCCACCTTAGTTCACTGTGATATGAAAACGTTTGATCATGTTGCTATAACTGTTGTGCCCCACGATAAACAAACTCCAATTGTGCCAACAGAATCTGGTTCT
ACTCCATTAAATTATGTCACACCATCCGACGTAATAGCTGGTATCATCAATGGATCGGCATTCTGTTATCAAGAAGTCAGCTACACCTGTAGAAATTCTCCTA
TTAATATAAATGGTGAACAAATTGTATCTGTAAGTGTTGGTAAGGAATCTAGAAATATTGACTACTTCCCTGGTGGAGAAGGTGTGGCCGGTAATTGTGGG
TGTGGTATAACTGGTTCCTGTGTTGAAAAGAATATAACGTGTAATTGTGATATGAATGACAATGTTACCAGATATGATATTGGTATGCAGACAAACCCTCAA
GACTTACCAGTAAGGTCAATTACCACTGATAGTAGCTCGAATGATTCTTATACTGAAGTTAAAATTGGACCACTGATGTGCGCTAAAATGCAGTTTGGTATT
GAACCGAATTGTGAAAAATATCGGGCAACAGGAAGAACTGAACCATACACCTACCTGATAGATCCGGATGGTCCTGCTGATCCTTCTAAACCTGATGACTT
AAATGTTGCACCATTTGCTGCTGAATGTCAATTTACAGAGTATCCACCACAAGGTGTTACTATAATACATCACGATACAGAAAATCCCTTCAACTTAACGGA
AGCCAACTTTTTCTTTGAGATAGTCTACAAGAGAATAATTAGTGATGATCAACTTAGACAGTTACGTACCAGATCAACTTATTGTGTACAGGAATTATCATTT
AACTGTGAAAATTATCATCTACATCTTCAAGGTGGGGGTGGTGTATTCTGGAAGGATGTTGATGGTCAGCGGTATGATTACTTGTCTGGTGGAGCTGAGG
GTGATGTGGGCTGCAGTTGTAACGCACTTCAAAATTGTGCTGAAGGCAAAACCTGTAATTGTGATGCTATGAGCAGTAGCCTTCAGACCGATTTCAATGAG
TTAGGAAATGTAACCCATTTACCCGTTTCATCACTAGACTTTACTTCTGTCAACGGTCAGCTTTCAGCTGGTGCCAACTCTAGTTTAGGAGTACTGTTAGGTC
CATTGAAATGCTATGAGACTTTCCCTACCTGCTATGATTTATGGGCCTACATGAGAACCAGATCGTTAATTGGAGAAACACCAGTAGAATCTGGTAAATACA
CCATCGATCCGGATAGTTCTGGTGGTGTTCCTGCCTTCACCGTAACCTGTAACTTTCCATTTACGTCAGTTCCTGTAGATGGAAGTGGTGGTGCTCCAGGAC
TCGGCCAGTCGCAATGTTTTGAAATCGTATATAAAGATACAATGGGAAACCCAATTACACCAGCACAAATATCGGCTTTAGTGGACAAATCGAAGGCGTGT
CAACAGTATCTTGGTTATGCTTGTATCAATGCTCCTAAGACCAAGAATGTAAATTTCACTACCTGTGATGGCAAGGCACAAGTTGGTTGGGCTTTAAGTGGT
GGATATGACAGCTGTTCTTGTGGCTTAACTGGTACCTGTGAAGGTGGACCACAGAATTCATGTAACTGTGATGTACAAGATGGTACCAAGAAAACAGACG
GAGGCTCTATCTACAATACGACACGATTACCTATCTGTGAAGTGTGTTTAGGTTTGGACAGCGTTGACACTCTCCCGACGGGTCAACAGACCAGGGGTAGT
GGATTTAGTGTCGGGACTTTGAACTGCCTTGGATATGACTTTGATGGAAAGAATAATTGTAATGATCGACGTCTAACAAGAACCGAGGATAAGGTATATTA
TCTAGATACTGATAATGCATACTCTGGTGAAAAACCTTTCCCAGTATACTGTGACATGGTACCATACCCTGCTATGGGTATCATGGAAATAAGGCCTAAAGA
GTCCGAATACCAAATCCCTGCCAACCAACCCTTTGATATCAGCATCACCTACTTTGTGTATAGCCAAGAACTAGTAAATCAACTGATAGACAATTCTTATTAC
TGTACACAAGAGATCTATATCACATGTAATAGTGGTTCTTTAGATTTAACTGGAACTAATGGATGGTATGCTAGAGATGGAACACCACAAGATGATTGGGG
AGGAAATATTGGAAAAACAGTAAAATGCCAGGACAATGGTGCCGACTGTAACTGCAATAAAGCCGGAAGTCAGATTGATGGAGGTATTTTAGGTGATAA
ATCTAAGTTACCAATATCAAGAGTTAAGCTTGGACCAAGCACCACGGCCAGAACTCTCACAGTTGGGGCAGTGAAGTGCTATGAAATCAGAGCTGACTGCT
ATGATATCATGAAGAGTGGTGATACTAAAACTCCATACAAGAACGGAACTTACGTCATTGACCCTGATGGTCCGGATGGCGTTGATCCATTTATTGTTAAAT
GTCATTTTGATCAAAAGGGGGACAACGCTGTTACTGAGGTTGTTACTGAGGCTCAAGGACCTAGATTTTTAGATGGAAAGACAGTACCATACTCATATTCC
ATACCTTATGAATACAAAGGAGCAACAGAAGAACAGATAAACGTTCTGGCCAGACAAGCTCCATTCTGTCATCAGGGAATCCTTTACGAATGTACGGCATC
ACCAATATCTGGTAACGTCAATTATGAACTAAATAATGGATTTATCTCACCAAGCTTTGGAACCGGTCCCGAGACCAATGTTAGTTCATGTCCATGTGATCTT
CTTGGAACATGTGATGGTTTCAAATGTCGATGCGACGCCCAAGGATCAATGGTAGCCAACGACAACGGAACAATCACAGATAAAAATCTTCTGCCCATTAC
AGAGGTCAGTGCCGGTGGCCAGACAGAGTCTGGTGCAAGAGCAGCCGTTGCCGTCTCTTCTGTTCAATGTTCAGAAAGACCTGTCGGGTTCCCCAAGGAT
TGCGAAGAGGCTCACAAACGTGGATACAAAACTGGAGAAGTTATGATATACCCATCAGCTTCTTTAGATCCCTTCTTCGTTTATTGTGATATGCAAGTAAAA
CCCGGTCACGGTGTGGCTATTATCAATCCAAACTTAGAACCAGAAACAAACGTTCCTTTTAATGGTCAACCTATAGATGTACCATACAAGGGACTCACGAAT
GAACAAATTCAAGCAATTGTAGATGTTTCGTCTAACTGTTACCAACCCGTGAAATATAACTGTAGAAATACAGCATTCATATCAGCTAACAAATTTTCATGG
AAGAGTATTGATGGTAGTAGTAATAGCTATTTTGGAGCTGGTTCACCAGCAGGAAGAGATTGTACATGTGGCAGCGAAAATCTTTGTGGAGGTCAAGGCG
GTAAAGCCACCATGCAAAAAAGGAACTGTAACTGTGGCATTGGAGATAATCAGGATCGAGTTGATGCTGGTATCTTTTCCAACACCAAAGAATTACCAGTA
AGGAGTCTTGTATTCTCACCTACAGGGAAATCAGGATCACAAGGAAATATAACTATTGGTCAACTATATTGTGGACAAACAACACTAAAATTCGATGAATG
TACAACAGGATTCCATGATTGTCATGAGTTAGCAAGATGTTTAAACACTGACGATGGATTTGAATGCGTTTGTCAACAAGGTTACAGAGGCCAAGGACGAT
GGAATGTTTGGGCGAACGGAAGAGAGTGCTTTGATGATGATGAGTGTGCTTTCTTACCATGTCCACATTCAGCTGTTTGTGCCAACTTCCCCGGTTCTTTTA
ATTGTACCTGTAAAGCTGGTTATTATCAAACAGGTCCCCAACAATGTCAAGACGTTGATGAGTGTGCTGAGAAAAAGGATAATTGTGATCCAAATGCTCGA
TGTACTAATACTGATGGTTCATTCTTCTGTACCTGTAAACGTGGTTATAGAGGAGCTGGTACTGATGGTACATGTGAACCTGTTGGAATCTGTGCCTGTTTT
GGCGATCCACATTGTATTTCATTTGATAATCATTGGCTACATTTTATGGGTGATTGTCAGTACGTTATGTCTAAAGATGGTTGTGATGGTTCACCACAAACGT
ACGGGGTATGGACACGCAACTGGGATATGGAGAGAAGAGATGTTCAGGGTATTACCTGGGTTAAGGATGTTACTGTAGTGGTTAATAACACGAAAATCTT
GTTAGGAAGAAATCGTGAGATAAGAGTAGATGGCAGAACTTTCATTGCTTATAGTGATAACGGTATTACTGTGGATGACAATGGAAGAGAAGTCGCAGTC
ACAACAACATTTGGTTTAGAAGTTTATTGGGATGGAGGAGATGAAGTAGAAGTTATATTACCTAAAGAATATAGAAGTAAAACATGTGGTTTATGTGGTAA
TTATAATGGTGAAATAGAGGATGATTGGGTTGTTGGAGATCATTGTGCTAAAGAGAAAGGAAATGTGACTGATAATGAAAACCTCTTCGGAAACTCCTGG
GTTGTATCTGAATATCTTGAAGCTGCAGCGGCATGTCAAGCTGATTGTGATGCCCCACCTACTAATAATACTTTATGTAATGAACCATTAGTCAATTATTATT
GTGATCGAGTATTTAATCCTGAAATATCCCCATTCAAGGACTGTCTAGCCGCAATGGATGAAAACTTACTACAAAACCTCAGATATAGTTGTGTATTTGATA
TCTGCCACGTGACACAAGATGTAGAATTGGGTATGTGTTCCAGTGCCGAGAGAGTTGTCAATGAATGTCAGAAAAACATCAATATTGATGTGGGCTTGTG
GAGAAAACCTGGATTCTGCGAAATAACATGTAGTGTAAATATGGAATACCGAATCTGCGGACCAAGAGGTGAACCTGATAAACAACGACAAACAACATGT
ATGAACGTCCGGCTGGGTACTAATCCACCTAACCTAGGTTTCTGTGCTGAAGGTTGTTTCTGTATACCGGGTTATGTTCTAGAAGGACAGAAATGTATTCTT

CCAACAGACTGTGGATGTGTTATGGATGGTGTCTACATGGCGGTTGGGGATACATATGTTGATCCTGGATGTACTAAGATGCAGGTCTGTTATGAAAATG
GAAATTTAACAGAACAGTCCCTTCAGTGTGGAGCTCTAGGAGAATGCACGCTAGATGATAAGACTGGAATTTATGGCTGCCATTGTAAGGAGGGTTACCTT
GGTGATGGATTCGAATGCTCAGATGATCCATGTTTTAATGTAAAATGTGGTAATAACATGCAGTGTGTAAAAGGTGTTTGTCAGTGTAAGATGGGTTATAC
CGGTGATTGTAATAACTGCGATGATATTGACGAATGTGCTACTAAAACTGATAATTGTATTCATCTTGGTCAGAGATGTAGTAATTATATCGGTGGATATGA
TTGTCTATGTTTAGACGGATATGTCAGATACGGAAATTACTGCAGTGATATCAACGAATGTGAAGCTGGGTTTGATACATGTGATGAACATGGTGAATGTA
TCAATACACATGGTAGCTATTTATGTGAATGTTGTCAAGGTTATACCAAGAATGCTCTAGATACCTGTGTCCCTGATGGCACCCCTGTTCCTGCAACCGGAA
GTAAATGCTGCTCATGCTCTGGACCAGCTTGTTCTAGACCAGGCAAGGTTTGTGGTGACGATGGTAAAACCTATAACAATGAAAGAGCTCTTCGAATTGCT
GCATGTAAAGCAGGAGCTGATGTAGATGTTAGTTACAAAGGAGAATGTAAACCTTCCTGTGGTGGAGTTAGTTGTGAGAAACCATTTGAGAAATGTGTTG
TTGATAACACAATAGGTGTAGCTCAATGTGAATGTCCTAAATGTGGAGGAGGTGATGTCACAGTAACTGAAGCGCCTGTGTGTAGCAGTCGATTTATCATG
TTTCCCAGTGAATGTGAATTCAAACGCATCATGTGTGAAGTAAATGATGCCACTACGATCATCCAGAGTGATGCCGGTCTTTGTGACAAAGGCCCAGGAAG
TAGCCCAGTTGGACCATGGACAGAATGGGGTGACTGCAGTGTAGATTGTGGTAGGGGTATTATGAATCGAACTAGAGAAGAAATCAGAGCAAGAACTAG
CTACGAGAAAATCTTGACGCCTATCTCAGAAACAGCAGCTTGTTATGGAAATTGT
>CL3399.Contig1_L1_2_1
TGAAATTCAAGAGTTGTTTAGCCTCTGGTGACTCCCTCTATAGAACATTTGACGGTGTTTATTTCAACTACAAGTCAACATGTGAAGTCACCTTGGCGAAAA
CCAACGACTTTACTGTGACGGCATCTACAGACTGCAATGCTGAATCAATCTGCCAGAAGAAAATCAAAATCACCGATGTTGTAAATAGTGCTACATTCTCTT
ACGTGGATGGTCAAGTATATATTAATTCCGTCAAATGTCCCGTAACACAAGAAGAACCTTATTCCGACTCACTTGTCAATGTTGTTCGATCTGGTGACTCGA
TTTACATCAAACATTCATCCGGTTCTACTGGACTTACCGTTCGAGTTGACAAAGACGGCGCTGCCTACGTCACACTGAGCAGAACAGGTCTTATTGGAAGTG
GTGGTGTAACTGGTATGTGTGGAGACTTCGATGGTGATACATCAGACGAGGCTGATCTCTCCGCTGATGGATTCAAAGCTAAAGCTATGGGAGATTCGCA
ATTAGTAGCCGGTGGTACATGTGGTCAAGCTTTACCCGAATGTTCCGAAACTCAACTATCAGTAGCCGCACAAGAATGTTCATACCTGAAGTCTAGCATGTT
CGCTTCGTGCCACGGAGTTGTCGATGTCAATTCCTATTTTGAAATGTGCAAAGAAGCTTACTGTGCAGCTGCTGTAGCCAGTGAGGCGTCAGCCAAAGAAG
CTCGTTGTAACAGATTTAAGATGTATGCAACCGAATGTGCTAATAATGAAATCGTCATCAGTTGGAGATCACCGCTCTTGTGTCCGATTGTGTGCCCAGCCG
GTATGGACTACAGTCAATGTGGAAGTTCATGTCCTCGCGGATGTGGAAATTACTTTGCTTCTTTGAACAAAGATGAATGTGGTCGATGTGTACCTGGTTGC
CAATGTAAGCCTGGTCTGGTCTTACACAATGGCGCATGCGTAACAGCAGATAAATGCTCTTGTAAATACCAGAAACAAGACTACAACTATAATCAAACCGT
AGTCTCCGAAAACAACTGTCAAACATGCACTTGTGGTCTCTATGGATATTGGAATTGCGTAAGCAGACCATGCAGCGCTACCTGTGAAATTGTTGGAGTTG
GTTCTATGAAGACTTTTGATGGAGCATCTATTCATATTGGAGCTACTACGTCTACTTGTAAATTCACCCTTGTACAGGGAGCCGACCTTGAAATATCGTTAG
ATACAACTAAATGCGTCAGTGGAATAAATGCTGTCTGTATTAAGGGCGTGAAAGTTGTTTATAAAGGTCAAGCTGTAGAATTGAAAAATATAGATTCCTCA
ACACGACTCATCACCAGCTCAAGTGACAACACACCAATGCCTTTCTCTGTTGGTAACTTCTACATTAAGAAGGTCACCAACTTGTACTACGTTATTGAAGGTC
CAAATGTTCGAGTCCTTTTTAGTGATGCTGGTCGATTAACAATACGATTACAACCGGCGTATGTAAATCAAACTGCTGGTGCATGTGGGAAATTTAATTACC
AATTCAATGACGAATTTGAGCTACCCTCAGGCGGTGCTGGTACATCCAGTGAATTTCTCCAAGCCTATTCTGATGGCAACTGCGGCGCTCATTTTGATATAA
GCACTGCCACCAAGACATCATCTGCTATGTGTAACGAGTTAGATACAATGTTTACTGTGAGCACATGTAGTAACACCCAACCGACAGACCGCGTTTCCGGTT
TAAAGGAACTCTGTGAAATATCAGCAGATCCATTAAACGTCTGTCCAATCGCTTTTGCCCTGATGGGAGACTGTGGTGTTACCAAGGAACAATTTGAAGCA
CAAACAAATCTGTACGCCAAGTGTAGTCAATACTTTACTTGCGAAAGTGGATATATCTTTGAAAATTGTCAATCGGTCTGTAAAAGTGCATGCCGTGATTAT
GCCTTGTCCACAACATGCATGGAACAATGTGTACCTGGTTGTTCATGCCCAAGTGGTGATCGGCAAACTGACAGCTTATCTGGAATGAACAGCTGCGCAAC
ACAAGCTAGTTGTACTTGCTATGACATTGCCAGCAATGAGGTCACAGCTGCCGGTACATCTACACAAAGAGGCTGCCAGGAATGTACCTGTTCCAACGCAA
CTTTATCATGCGTCGACTCTCAATGTACAGATGTAATATGTTCTTCTGGTTTGGAAAAAGTAGATGCTACCCCAACTGAATGCAGACCACGATGTCCAAACA
AATTTGAAGCTAATTCTACATGTGCCGAGCAGACATCTCTAACATCAAGACGATGCTTTTGTCCCGTTGGACAATGGGAAAATTTGGAAGGAAAATGTATA
GCCCCTGAAACATGTCCCTGTTATCATATGGGTAACTGGTATGAACATAACAGAGCTATTGATTATAACTGCAAGACACTACAATGTAACAACGGTGTTTGG
AGTCAGACGGCTGTACAAGAATGTTCATCAACTTGTTGGGCATCTGGAGATCCCCATTACTCCACTTTTGATGGCAAAACATTCAAATACCAAGGAGATTGT
ACTTACAACTTCGTTAAAACTACCACCGAATCACCTGTTCCTGTTCAATTTGATATCAAAGTTGAAAATGTTGTCTGTGGTTCCACTGGAGTGTCATGTACTA
AAGCTATCACAATTAACGTGAATGACACATTGATTACAATGACACGTGGTGAAGATATAAAGATTGGAACAACACCATACACACCAGCCATGGGATTCCAG
CAAATTTCTATTATTACTATCTACAACGCTGGTCTCTTCACATTTGTTGATTTTGAAGGTGTCACTGCTATGTGGGACGGAGGAACCAGATTCGCCGTAACCT
TGACCAGTGCATGGAAGGGTAAAGTTAAGGGTCTTTGTGGTAACTTTGATGGTAATATTGACAATGATTTTACCACTGAAACTGGAACGCTTGCAGCAAAT
GAAGTTGAATTTGGAAACTCATGGAAGGCCGGAAGTTGTGGAGATGTTACTGAAGCACCACAATTACAAGAAGCCTGCATGACTGTTGATAACGTACCTA
GTCCACGAACAACTTGGGCTAAGAACCAATGTGAAGTTATTCTTACCGGTCTGGTCTTTGAGAATTGCCGAAATGTCTTAAATGATGAAGTCAACAAATATT
ATACCGATTGTTTGTATGATGCCTGTGGATGTGACCGAGGTGGTGATTGTGAGTGTTTGTGTACAGCCATAAGTAACTTTGCTGAAGCCTGTAACCGTGCT
GGTCAACCAGCCAGATGGAGACATCAACGTCTATGTCCAATTCAGTGTGAGTACGGCAGTGTGTACAAACCATGTGGCTCCGCATGCCCAAAAACATGTAT
GGATCCTTACGGTACCAAGTATGGATCGTTCTGTGTACAGACAACATGTGTTGAAGGTTGTTTCTGTCCCACCGACTACGTCATGCAAGCTGGAAGTGAAG
TTAAATGTATTCCACAAACCGAATGCCCATGTACATACCAGGGAGTTACATATAACAATGGTGACGTAATCACACAGGATTGCATGAAATGTACCTGTGTT
GGTAGTACGTTCAGTTGTGATGGAGCAAACTGTACCCGTGAATGTACAGGAGATGAATTCAAGTGTCAACAGGATGGATACTGTGTATCCAAAACCCTTCT
ATGTAATGGTATCCCAAATTGTAATGACAGATCAGATGAAGTTGATTGTGTCAAAGAATGCTCTACCGAGGAATTTGAGTGTTACGTCAATGGCCAGTGTA
TACCTCTAAATAATACTTGTAATGGTATCAGCAATTGCGAGGACGGAAGTGACGAATACAATTGTGCTCAAACTCGCACCTGTTCCGGTTCCGAATTTACCT
GTACCAATGGAAAATGTATTCCAAAGGCCTTTAATTGTGACGCTGCTAATGATTGTGGCGATTTTTCAGATGAACAAAATTGTCAACAATGTAATGAAGGT
GAAGTTATGTGCTCAGCCACACCTTTAATGTGTATTGCCAACAGACTTTTATGTGATGGACATGACGACTGTGGGGATGGTACAGACGAAATCGGATGCAC
AACACCAACGGGCATGACAACAACCAAAGTATGTGACAAGATAAATCTTGATGCTTCCACACCTGATTTATTATTGGTCGGACGAATCAACAGTACCTCAC
AGCCCTCCGTCACCCAAGCTCTCAAACCCAGTGGTTCAAACACATCCACCGTTTGGAATCCAACCGTTGGTGTGACAGCTCAGTCAGTCATAACCCTACAGA
TTGAGCTCGCCGTTCCACCGGAAACCCTCTGGACTGTTTACGAAGTTAGAGTATCCATCCGAAATGCTGCTAGTGTGACCATCGAATACACTATCACCGCTG
GCTCATTTATAAAACCACAATCCCGTGAACCAAACCCTGGAAAAGATTTCATAACCACATACAGCACACCTAGTGGTATGGATGTTTCCAGGATTGTGATCT
ACATTACACCGGAATCGAACGCAACCAGTATCAGTGTTTGGGATCTTTCATTGGTCGCCTGTTATGAAGGACTTACAACCCTGCCAACGCCAAGCACCACTC
CAATGGAAACAACAACTGGCGGTACAGAAACTACAACTGGTATTACTTCCACCGTCGTTTCAACCGTTTCAACAACAGAACCCTGTTCATCAACTCAGTATT
ACTGTAACAGTGTTCAAATCTGTATTATGATGAATCAAACATGCGATGGTGTATGTGACTGTCAAATTCCATCTTCAACTGAATGTGAAGATGAAACATTGC
CAACCTGTGTCACTACCACATCAACTAGTACCGAAGCCACAA
>CL6799.Contig2_L1_2_1
AGGAAAGCGTGCAAGCAGAAAAGGCAGACGAAAGTTTTCACGCAGTAACCGATCACGCAGAAGAAACGGTTCACGCAGAGGAAATGGTTCAAGCAGAG
TTGGTGGTTCTCAAAGGCGTAGAAATAAAAGAGAAATATATGGAGATTTCTTGACAGGCATGTGTGGAAATTATGGGGGATTTAATAGAAAACAATCTAG
AGTGTACTTGGCATATTTGAGCGGAGAATATATGCCCAAGGCATGGAAGAATGGAAATGATCGTAATGGTATCACTGATTGTTTAGCTATTTATATTTCATC
AAGCGGTATAACCAGGATGAAATTGGCATCCTGTAACAGAAAGTACGCATTTATATGTGAAAGAAACGGACAGGTTGATTCCAGTCAAATATATGAAACA
GAATGGTTAGATAGTGGTAAACCTGAGAATAGAGAGGATGAAAATGTTATAACCTTCATAAACAGAAATTTGCCTTATACAGGAAAACGACCATGTTCCAA
TCCAATTAGTATGAAATGTAGAATAGTCGGTACAAAGACACTGATTTCTAAAGCAAATAGTAATGGTTTTAAGTTGGAGAGAGGCAAAGCATGTGAGAAT
GGTCGGTTTAGATGTTTGGATAGAAAGAATGGTGGTAAAAAGTGCCCAGACATTGAATATAAACTTACTTGTCCACTTGACAATAATGATTGCAGTTTACC
CAGAAATCAAGAAAACTGTAGAAACAAGAACATGAGATGTATGAATCGAGCTAATGGTGGCTACTCATGTGTCAATCCATGCAAGAGACTCGACAAGGTC

TGCTTTGATTATTGTGAAAAGGCCGCTTTATGTAAATCAAGTGGTGATCCTCATTATATGACATTAGATGGAAAGAAATACAATTTCCAAGGCGATTGTTCA
TACACATTGATTCAATTACGTCCAGGAATACAAGGTCAAGAAGCATTTTCAGTTAGAGTTAACAACTATAAGAAGAAACCTAGTGCCAAAGTCTCCTACACC
GATTGGGTCGAACTTGACTATATGGGTCATAAATACAGAATTGGAAGGAAAAAGAAAATATATGTTGACAATGAAGAAACTCTTTGTCCTTTTGAAAATGA
CCATGTGAAAATTAGCTCCTGTGGTGATAGAATCACCATTTCGACTGATTTTGGTTTGGAATTGTCAACAGATGGCCGCAAAAGTTTTTATGTTGAAGCATC
ATACAAACTACAAAACAAAGTAGAAGGTTTATGTGGTAACTTCGATGGAAACCCTGAAAATGATTTACAGATGGTTGGAGCACATGCTTATAATAAAAAGC
TTACTCCGGCTGAAAATTTTGCCAACAGTTTTGCTGCTGATGATCATGAATGTAGACTACCAGATACAGATACAGAACCTAAATGTTCAAAGGATGATGAAC
GTAGAATCAAGGAGGATCAAAAGGATGGCTGTGGATTACTCAAAAAGTCATCCAACTCTGTTTTTATTAAAGCTGCCAATGGAAACCGAGCTCTTTTGAAA
ATGTTGAAAGAGAAATTAAACAGTTGCGTTTATGATCTTTGTCTTTCTGAAAATACCAGATGTGATTATTACCAAGCTGCAGCAGATGAACTTAGTAGTATT
GGCTCAGGAGTACCAAACTGGAGAAAGGAAACTAACTGTAAACTGGAATGTAATGGTGATTTAGTACCAGTATCAAATCCACCACTTAAAGAAGCTACAT
GCATGGAACCAATAATAAACAGTAATGACAAAGAAGTCGGTGAAGTGTGCCGATGTCCAGGTGATAAAATTAAAAGTATCGATGATAAATGTGTTGATGT
ATCTGAGTGTATGGGTTGTGAATTCCGTAGGCAATTCATTCCGGTTGGTCGTCCTGTCCATTCATCAGATTGTAAGATGACATATCACTGTCGAGTTACAAA
AGATGGTAGTTCAACAAGTTTAATGTTTGAACCAAGAACATGTGGAGAAAATACTAAGTGTGAAGGCGGTAACTGTGTTTGTATAGCAGGCTTTGTAATGA
AAAATGAAAAATGTGAAGAAGATCATAGTTCTATCAAATGTGCTCATAGCTTTTTAAAGAAAGGACGCAAATGTTACAAACTTTTGCACAGAAAAGCAAAT
TGGCATGAGGCTGCGGTGGCTTGTGCTGGACAAATAGATGGCGGTCGATTGGCTAGATATGATACAGATGAAGATAAAACCTTCTTAACTGAACTAGCTT
CTAGAATGTCAGGATCTTTAAGAGCTAAAGAATTAAGGATGCAATCTAAGAAAAGAGGAAGACGTCGAGGTGTTTCCAGTAGCTATGTTTTCTGGATGAG
TGGTAATACAAATAGATTAGATCCAAGATTTAATCCAAATCCATACTCACAACTAGATGTGCCACAATATCTTGATGAGTTATCTAATCGAGTTGGTTGTGT
ATATGGTGAATTTAAAGTCAGAGGAAAGGGGCGAAGGCCAGCCAAGAGATACAATAAAAACTTTAAGATAAGATCAAAGAGACGATCAAGGAGAAACA
GAAACAAGGCACGCAGATCAAGGAGAAACAGAAACAAGGCACGCAGATCAAGGAGAAACAGAAACAAGGCACGCAGATCAAGGAGAAACAGAAACAA
GGCACGCAGATCAAGGAGAAACAGAAACAAG
>Unigene33579_L1_2_1
CCGAAAGGAACGTAGTTGAGATTATAATAGACCCAACCGCACCTCTACTAACAAAGAATATCACACGCTCGTGTTGACTATAAATTCCACGAATTTATATAA
CAATCCCGAAAAATATTGACTGCTCAGTCTGTGGTAAACAATTTTATTGAATTGTAAATTGTAAGATTTATAAGCAGTAAATTGATTGTCCATTGACAGAAG
TGACACGCTTTATTGTGTAAAATATTTATAAAGTTGTGCGATTTGAGCGATTTTGGTCATTGGCACGGGCCACCCGTTCAACAACTACAGAATGGCGAGTAA
TTGGAAAGTTTTTCTTTCAATCTTAGCCTCTGCGCTAAGCTATTGTCTATGTGCAGAACAGGGAACATATATGGTGCTTTTACCTAAAACTATTCGCCCATGT
GTTGATTATACCTTCACGGCACAAGTTCTAGGAAGTCAGCATTACAACCTTACAACTACATTAGTGAATAGAGCAGGGACGGTATTCGCAGAGTACGGCCC
AACATTGATACGTACAACCCGAACGATTAATCTGAAGATTCCGTGCGATTTAGTACAATCATTTTACAGCATTAAAGTTGTGGGAACTGGTGGCGTAGAATT
TGAAAAGACGAATTATGTTACCTTTAATAGCAATAATTTGGAGATTGTAATACAAACAGATAAAAAGGTTTATAAACCTGGGCAAATAGTGAAATACCGTA
TTTTTGGTGTAAAGCCCAACCTTACCCCTGAACGGGGCAGTTTTGAAGTAACAGTTACGGATCCACGACGAAATAAGATTCAATATTACAACAAAGAAGGG
GATGGTTATGGTGTTGTCGAACATTCCCTTCAACTATCAAGTGAACCCATCTTCGGAACCTGGTCTATTAGCGCAGTTGTCAGTGGTACCAAACACACAGTA
ACCTTCACCATTGAAAAATACGTTTTACCTAAGGCTGAAGCCAAAGTTTCACTTCCCTCATTTGTTGCTGAAAAAGATGAAGAAGTCATTGTTAAAGTTTCAT
CAATGTACACATTCGGAAAGCCTGTGAGAGGATCTGTAGTATTGCGAGTAACGGTGGACTATTACAATACAAGAAAATATGTACGAAATCCAGAAACAGG
AAAATATGAAGTGCAGATCATCAAACAACCTGAACTTATGTATGATTTGCAGCTGAATGATAATGGTGATGCTGATGTTCGAATAACTTTAAAGGAATTGT
CGACTATCGCTAGCTACAACAGTATATCCTATAAAACTATTGTAGCTCAAGCCAATGTTACAGATAGTGAAACAGGAATTATGTATGTCTCAAAGCCTTCCA
AAACATCCGTGAAGAACACCCGAGAAAAAATTAGCTTTGCTCTGAATCCAACAAACTACAAACCTGGTCTGACAAATCATCTTTTTATCGACTTGAAACAAC
AAGATGATCGACCAGTCCTCAACCCTAAAACTATTAAGGTTACTGGAACAGTCCAATTTGAAGTCCCTAAAACCACGACTACAACTACCACTACCACTACGA
CAACTACTACTCCGAGCCCAAACGGAGCATTAGTCGAAGAGCCTATGCTAGTGAAAGAAGTTATTGCTCGTCCATACTATCCTATTTGCGGAGGTCCAAGTT
ATGAATATCGCAATCTTGAAGAACAGATACTGGAAGTGCCAAGTAATGGATTGGTTCCGTATGATCTGCTGGTTACTGATTTAAATGCTTTGACCGTCTCAT
TAACGGCGACATATACCGGTGATACCCAAGTTACCAGAACCCAAAGTTTATCTGTAACAAGATCACCCAGCAAAACCTTTATATCAGTGACGCTGAGAACTT
CTAAACCAAAAACTAATTCCAACATATTTTTTGATATAAAATCCACTGCTTCGTTTAAAAAGGTTTACTATACGATCCTATCGAGGGGTGTGGTGGTATTTAA
TAGGTACCGGTCCTACAGATCAGCCAGTAAAGAACAAGAATTAAGAGTTGCTGTACGATACAATATGGCACCCAAATTAAGAATAATTGTCTTCATTCGAG
GTGAAAATGATGAAATCGTCGCTGATGCTATATCATTTAACGTGGATGGACTCTTCAGAAATAAGGTTTCCTTGAGTTTCAATAAAGACAAAGTAGAGACC
GGTGAAGAGGTAACCCTTGGAGTCAATGCTTATAGATATTCACAAGTATATGCTCTGGCTGTTGATAAAAGTACAACTCTGCTGGGAGCGCCTGCTGAAGA
TCATATTTCCAATGTTCAAGATTTCCTGAATCAACTGGATACAGGATATACACCATATTATTACGGTGGTGGTGGAGGTGGTTTTGAAAGACCCATGATAGC
GTTCAGAAAGAAACGAGCAGCTGAACTTAGGAAGAAGAGGCAAGCCGAACTATTGAGTCGTCATCGCCGACTATCAATATACTGGCCATATCCTTACTTTA
CATCTGGTGTTGATGCCGAGGATATGTTTAGGAATACGGGAGTTTACGTAGTAACCGATGCTTTCATTTACAACTATGTAGCACCTAGATCTACTTGTTTAT
ATCCCAGCTATTACCTCAGAGGCGGTGAGCCACCGATGGCAGAAATGAAAATGGAAAGTGCTGTACAGGCTCCACCGCTTGCAGTAGAAACATTAGATGT
TGATACACCTGTGCGATCTTTTTTCCCAGAAACATGGATCTGGGATTCACAAAATGCTGGTGTCACTGGTGTAGCTAATTTTACACTGAACGTACCAGACAC
AATAACAACATGGACTGCCAGCGCATTTGCTATAAGTAGATATCCCAGTATTGGTGTGGGATTAACCTCTGACATAGCGTCGATTACAGTTCTTAGACAATT
CTTTGCTAGTTTAAATCTTCCATATTCTGTTATCCGAGGTGAGGAAGTGAGAATCCAGGTGACAATATTCAACTACCTTGGAGATGACACTGAGGCTCAAGT
TGTCTTAGAAAAAGCCTCTGGTTATATAGTTCTAGAACTGGACAGTGAGGGGTTCCAGTATGAAACACAAAACGCTAAAATTCAAACCATAAAGGTAGCTG
CTGATGGAACTGCGTCTGCCTACTTCACAATTAGGTGTACCCAAGTTGGAACAATAAACATTCAAGTTGCTGCCAAAGCTTCTGGTAACCAAGCCGGTGAT
AGGGTTTCCAGACAACTTTTGGTCGAGCCCGAGGGTATACCAGCTGAATATAATGCAGTGGTTTTAGTCGACTTAGAAGAGAAGAACCCGTTTGAAGCAA
GCATTCCTATTACTTTTCCACCAAATGTGGTTCCTGACTCTCATAGAATTATCATTACCGCTTTCGGAGACCCACTTGGCTCTGCCCTCAATAATCTTGAAAAC
TTGATCGGAATGCCTAGTGGTTGTGGTGAACAAAATATGTTGCGATTCGTCCCTAATATTTATGTTCGAATTTATCTGGATGCCACAGGTCAGCTGACAACC
GCCCTGAAAACCAAAATATCCGGTTATTTAGAATCAGGTTATACCAATCAGTTGAGGTATATGAGATTGGATTCTTCCTTTAGTGCTTTTGGAGACAGAGAC
AGCTCTGGTAGTCAATGGTTAACCGCCTATGTACTGAGAACATTTGCTGAAGCCAGTGAATATGTATATATTGACGAGAATATATTAGGTAGAGCAGCACG
TTACTTGATCAGTAATCAACAAGAAGATGGTTCCTTTATAGAACCCGGTCGTGTTATACATAAAGAATTAAGGGGAAGTTCTTTCCAAGGAGTAGGTTTGA
CAGCTTTCTCCTTACTAGCTCTGTTGGAAGTGAAAGACAAAAGTTCCATTCAGAACAACATTGACTTGACTATCAGTTATTTGGTGACAGCCTCTGCTACAAT
GACTAATGCTTACGAACTAGCCATCACTGCTTATGCTTTATCCAAAGCCAAAAGTCCTTCTGCTGACGCTGTTATAAGTAAATTAGAAGCTGCACAAGTAAC
CAAAGACGGTCACGTGTACTGGAATGTTGTCCTGGACGAAGATCGTGTGAAACATTTCTGGAACCCACCAAGGAAACAGAGCAGTGCCGTGAATATTGAA
GCTACAGCTTATGCTCTGTTAGTCTATGGACTAAAAAAAGATACACTCAAAGGTCTTCCAGTTATGTCATGGCTCATTTCGCAACAGAATGGTAATGGTGGA
TACTCTTCCTCTCAGGATACTGTTGTAGCCCTGTGGGCCATGTCTCAGTTTGCCCAGCTCATCTACGCTGATACATGGGATGTGAATC
>CL6346.Contig2_L1_2_1
GAAACCATGTCGGAAAATCCAGATGAACTTTCTCGGCCAATGCAGGGATCGAACACCCGACCTCCAGGCTAGCGAGCCAGCGTCTTAACCACCTAACCGC
GTGTAGGAAAGGCTTACAATAGTATCAAAAATATTATGCTCATTGATATCGAAACGTCGTAATTATAAAATAAAACCAGTGATTGTTTTCCATAAAATTTGT
GTTCTGTTAATGTTAGCCGGAGGTCCCATATATATCGAGGCCCTGTTTCAGTTTTCCAAGTCTATAGGTAAATCCGGCACTTATAAACTGTTGTAGCATCGCA
GTTTGTGTCCCATCAAAAGGTTTTACTAAGCGTGCGGACGTAAGTTTAGGGGATATCCGGAGTTAACTCATGTCCCTCGCAGTGTTTCGTTTGATTATACCT
GTACCAAATACCCCTCAGGTATATGGAGTTACCTGAGCTTGGTCCAAAATATCTAATGTCGACCTACCTGTATCATTACATTTATCACATCCACAAGATGATA
TTTCTGGTATTTGTACATCTCTGTTTGTACCTCCACAGTTAAACGTGTATGTCTTCGAACCAGTGTATGTTCCAGTGCAGCACTTACAGTTCTTGGTCATGTTA
CCAGAGGCGAAGTCGGGTATGAAAGAACTTGGTCCGCATTCACCCTCGCAATAATTGTATGTGATGTTTTGTGTTGAAACACAGTTTTCGAACGAGACTTC

ACCGGAGAAGACTGCCATTGAACAAGTCTTTGGTTTACAAACAGTTCCACAACATGGGTCGGGTACGAGGCTATGGGTGGTCAGATTACAAGTGCTGATA
TCGTCGCATGCTTTTGGATAGTCTATGATCTTTCCGTCCAAACATTGGTGACTTGTGCAACCATCGGCGTCCTTCCATGTTTCATTAACTCCATATGTGACACC
ATTGAAAACACATTTTTCACATTCAACAGGTTCAACACATGATCCATCCAACCGTCTGTAGCCGTCAGCACATATACATGTAAATTCTGGTGTTCCTGTCTCG
CAGTTATCTGGTGGTGTATCCATTCCACATGTGTATTCACAAGCAGTGAAGTTGCTGGGTTTCTCATTAGGTTCGGTACAATTGCAGTCCTGTAAGAAACAC
GGCTCGGTCTCTGAGGCGGGTGTAGATTGGACACATTCGGCACGAACAGTTCTTGTTCGAGTGTGTGTTCCCCATCCGCATTCTTCTGGTACTGTTTTGCCG
TTGAGAGAACATTGAGACCATGGTCCCCATTCTGTACCGTTAACACATAGACATGGTTCGTTATTACATTGTTCTACTACTGGGTCAATGCTGAGACAAACA
TTAGCTTCGTTTTGGTTGACCAAGATCTTGGGTCGTGATCGTGTTCCTACTGTTCCATTGCTTGAGCAGTTCTGGCTACAAGGTGTGTAATCGCCAAAGATG
AAATCACAGCATGGATCTGGACCACATAATTCTGTTTCTTGTGCGTCACCAACACATGTAGCTCCGTTACATTCAGCCTCGGGTGATCCACAGATTCGGTAT
CTGGTCTTGTTGTAATTTCTGCAGTCCTTCGAGCAATTACTCCATTGTGTCCATGTTGACCATTGTCCATCAACATCTTGTACAGCATCTGGACCACAATCAA
GGGTTCCATTCTTACATTCGCATTTTGTGCAGTTTTCAGAAACAGATTGACCATTTGGAAGTTCAGTACCATTTTCGAGAACACATGGGCATGGGCTCTGAC
AGGGTTCAGTCTCAACATCTGTTGGTTTGGGACATGTTGGGCTGTTAGAAGTTCTGGAACGTGATCGTACACCTTCTGTGCCCACAGAACAGTTGCTGACA
CAGTCTGACCATACTGACCATTCGCCGTATACACAACAATCTTGGATTGGTGTACATACGTATCCGTTGAGCATATCACATTTGCATTCTTCACATGTTTGTG
TGTTATTAGTTGAAAATCCGACTGGGTATTCGTTACCATCACTGTCGAAACATGGACATTCTTCTCTTTCCAGACACATTTGGTTGTATGTGAATCTATTTTCT
TCACATCTACATGCTGGTAAGCATGTTTCATTGTTGTATACTTCTTGTGGTGTTGATCCTAGTCGCATATCTCTACATGATTGTACACTTGGTAATGAACAATT
ACTGTTGTAGAATTGGTTCGTTTCACATATGGGTGTTGATGGACAGGAAACTGCACAGTCTCGGCTTTCAGATTGAAGGGCTTCGGAACATACACAGCCAG
CAGTATTATTACTACATGTCCTGCTTCTTGTTTGAGTCGTCTGTAGGCAGGCTGAAGTTGGGCAGTTTGAAAATTCACTCCATGTACTCCAGCATTCGGGTG
TTGGTGTTGAACATGGTTGTTCATTACATGGCTCGACTTCTGGTGCTGAACAGTTCGATGAGCTACCAGCGGGTTGTGCATTACGTGTTCGGGTTCTTTCTC
CGCCTCCACAGTTCTTTGAACAGTCACTCCATGGTGCCCATTCTGGACATTCGCAATCCATGACAGTACATTTTAGTGTGCCGTCCATTTCACAAGTGCAGTT
AAGGCACTGACCAACGACAGTAACTCCTGATGCCAACATCTGTCCATTTCCAAATCGTACTAAAGTTTCGTTATTCTTTACAACCTGACCTTGATCGTTGACC
AAATAACCCAACTCGTTGTTAGCCGGCAGGAAGGTCAGTTCAGCAATAATATCAGCTTCGTTCAAACTACATTGACAGCTATTACATGCTTCGGTTTCGACA
TCGGGAGTGCTGTTATCTCCACAACATGTGGCACAATTCCTTGTTCGTGATCGTGTACCGTTCATGCATGGTGTACATGGTCCCCATTCTTCCCATGGTCCGA
ATTCCCTTACACAGCCTGTGGGTGATGATGGCGTTCCTTGTGTTGCTGGTAATGTAGGTGAAGTAACTGCTGGTGGTCCTGTAGGTGAGGTTCCTGCTGTA
CCAGCTGTTTCTTCGAAGCAGGCTCTGACATCAATCTTGAAAACAGTAGGTTGGTCTACAGATGTTGGTGTTACTTCGATTCTGATATTTTCGGCTACTGTTC
CAGCTGGGAATGAGGCTTTAAGGCTAGGTAATGATAAATCAGCTGGGTTCAAGTACGTAGCTTCTGTTTCTGAACCGCCAGTTGGAGTTACAAATACTCTA
ATAGATTGAACATTATCGGGCGATACAAGCTGCACTGATTTAAGTTCTACCTCTTCATTTGTGACTGGTGTCAAATCAATTGTTACGTTTGGTGTACTTCCAG
CAGGGAAAGTAGCTGGGTCTGTACTTGCTGGTCGCAGGTTTTCTACATCAGCTGCTGGCACAGTTGTAATACTTGAAGCAGGGATAATAGCAGGATTATCC
ATTCCATTTACTTCAGCACAGACAACTGTAGGTGAGGTTCCTGCTGTTCCAGTTGTTACTACGAAGCAAGCGTGTACACTAACATCAATATTAATTGGTTCG
GTAATGTTTGTGGGTGTTATCTCTACCAAAATATCTGTGACAACTACTTCCGGTGGGAATGTAAGAATACCGTCTGAAGGATCATCATCAGTAGATTCAAGT
AGGGTCCATGGTGCTGAAGGTCCCGTTCGTCCAAATACTCTAAATGTCTTGACATTTTCCCTGACTGGAAGATTTACTTCTGACAGATCAACGGGTTCACCA
TCAGTAATTTTCAATGTGACGTTGGTAGCCGTGTCCGATGGGTTAATTGTAAATGGATCATCGCCTGGTCTCAGATCTGCTGGGTTATCTGTCGATGGGAA
GGCAGGTTCTTGTTCAAGGTAAGTATCAGGTATAACTGTTGGGCTTCCCATTCCTTCTGTTACAACACATCGTGGTGTAGTTGATCCCAAGGTTGGTGATGT
CCCAATTGCCACTGTAGGTGATGTTCCAATTGTCACTGTAGGTGATGTACCGAGTCCGGCTGTTTCTTCAAAGCACGCTCTGACATCAATCTTAAAAGAAGT
AGGCTGGCCTGAAGAGGTTGGTGTTACTTCGATTCTGATTTTTTCGGCTACTGTTCCAGCAGGGAATGAAGCTTTAGGGATAGGTAACGATAAATCAGCTG
GGTTCAAGTATGCAGCTTCTGTTTCTGAACCGTCAGTTGCAATTACAAATACTCTAATAGATTCAACATTCTGTGGTGATACAAGCTGCACTGATTTAAGTTC
TACCTCTTCATTTGTGACTGGTGTCAAATCAATTGTTACATCTGGTGTACTTCCAGCAGGGAAGGTGGCTGGGTCTGTACTTGTTGGTCTCAGGTTTTCTACA
TCAGTTGCTGCTGGCACAGTTGTAATACTTGAAGCAGGGATAATAGCAGGATTATCCATTCCATTTACTTCAGCACAGACAACTGTAGGTGAGGTTCCTGCT
GTTCCAGTTGTTACTACGAAGCAAGCGTGTACACTAACATCAATGTCAACTGGTTTGGTAGTGTCTGTGGGTTTTATCTCTACCAAAATATCTGTGACAACT
ACTTCCGGTGGGAATGTAAGAATACCGTCTGAAGGATCATCATCAGTTGATGTAAGTAGGGTCCAAGGTGCTGAAGGTCCCGTACGTCCAAATACTCTAAA
TGTGTCGACATTTTCCTTGACTGGAAGATTTACTTCTGACAGATCAACGGGTTCACCATCAGTAATTTTCAATGTGATGTTGGTAGGCGTGTCCGATGGGTT
AATTGTAAATGGATCATCGCCTGGTCTCAGATCTGCTGGGTTATCCGTCGATGGGAAGCCAGGTTCTTGTTCAAGGTAAGTATCAGGTATAACTGTTGGGC
TTCCCATTCCTTCTGTTACAACACATCGTGGTGTAGTTGATCCCAAGGTTGGTGATGTCCCAATTGCCACTGTAGGTGATGTTCCAATTGTCACTGTAGGTGA
TGTACCGAGTCCGGCTGTTTCTTCAAAGCACGCTCTGACATCAATCTTAAAAGAAGTAGGCTGGCCTGAAGAGGTTGGTGTTACTTCGATTCTGATTTTTTC
GGCTACTGTTCCAGCAGGGAATGAAGCTTTAGGGATAGGTAACGATAAATCAGCTGGGTTCAAGTATGCAGCTTCTGTTTCTGAACCGTCAGTTGCAATTA
CAAATACTCTAATAGATTCAACATTCTGTGGTGATACAAGCTGCACTGATTTAAGTTCTACCTCTTCATTTGTGACTGGTGTCAAATCAATTGTTACATCTGG
TGTACTTCCAGCAGGGAAGGTGGCTGGGTCTGTACTTGTTGGTCTCAGGTTTTCTACATCAGTTGCTGGCACAGTTGTAATACTTGAAGCAGGGATAATAG
CGGGATTGTCCATACCATTTACTTCAGCACAGGCAACTGTAGTTGACACAAATGGAGTACTGCTTGGTGGTGGAGCTAATGTAGTAGTCGTTGATGGCTGT
GTTGTTCTCTTCTCGGTAGTAGATACAGTTGAAGTAGAAGATAATATTGTACTAGCTTGGGTTGCTGTAGGAGAAGTTATTTTTTCGGTTGGGGATGTTGG
GGCTGTGGGAGAAGTTCCGGGCACAGTTGGTGAAGTTCCGGGTCCAGTTGGGGAAGTTCCTGGTGCTGTGGGAGATGTTCCTGCTGTGGCAGTCTCTTCA
AAACATGCTCTTACATCCAAATTAAATTTAACAGCAGCTGAAGTGATGGTTGGCACAATAGTAACTCGTATCTTTTGAGCTTTGGTTTCTGGAGGGAAATCA
GCTTTTGGTATCGGGTCCTGGAGACCAGCTTGATCTGTTACAGGAGTTTCATTGCCCTGTGAATCAATCAGCACTATTTCTATGGATTCGAAATTCTCAGCAT
TCGGGAATGTGATGGATCCCAGATCGACATCCTCATCGGTATTAGGTGAAAGATCTATGGTAAATAGTATAGGATTGCTGCCGTTGGGTATGATAGATAAT
GGGACATCGCTTGTCCTTAGATTATCCACTTGGTCAGGTGTCAAACCCTCAACACTTGAACTTGGTATAAATGCTGGTTTCTCCATTCCATTTATCTCCTCGCA
TCTCTCTGTGGGCGAAGTTCCGGGTGCAGTGGGCGAAGTTCCAGGAGCTGTGGGAGAAGTTCCGGGCACAGTTGGCGAAGTTCCGGGTGCAGTTGGTGA
TGTTCCTGGTGCTGTTGGCGATGTTCCTGGTGTACCAGTTGTCACTACGAAGCAAGCGTGTACACTAACATCAATATTAATTGGTTCGGTAATGTTTGTGGG
TGTTATCTCTACCAAAATATCTGTGACAACTACTTCCGGTGGGAATGTAAGAATACCGTCTGAAGGATCATCATCAGT
>Unigene11734_L1_2_1
GTAACGTAACATTTATTTCCAATTAGAAGTGAACGTACGTACAAAATTCTATGCAAAAACATTTTGAAATATAATTAACCGTACACTGGTATTCTCTTGTCGG
TAGACATTACTTTCACGTGTAGCTCATTCCAAAAAAATTAAAGAAAATAGTATTTAAAAACAAAAAAATAAAGTAGATATGGCCATTTCGTTGATGAATTCA
GATGATAGGATTTGGTGGATGCTTTCTAGTCACATGTGGTAACGATAATATATCTGATCCAACCTCGGAAATTATCTAAATCTGGACCACGGCCCACTTGGA
AGGCACACTGTGATCTCACAATGGTTTGATTGAAGGGTTTGTCGACTGTATTATCATTGACTGTACCAAACAGTTTAGTTGCAGCTTTATTATAGAAGAATT
CTACATCGCGCCAGTCATCTTGGGTATTGACTGACAAATCAGTAGTAGTTCTTGTCAAAGTTGAATCAAAGGTAGCTGCCCTGAGGTTAGTTGTCGGGCCA
GTCCTGGTGATTTCAATGGTAGCCAATTCGCCACAGTCTCCATTGGTGATCACTGCTTCTTCATTCGTGCGCCGAATGGGGGTGCTATAATCTACCTTGTAG
GACACTTTGATTTTCAGATTTCCCATGAACTCCACGTTAGACAAGCGAAGGATCCTGATAGATGTGCTTCCATTAAAATAGGCTGTACCGGCGGCGGTGTC
GATAACAGCGCCATAGTTGTCATACTCTCCGGCCTCAGCGGTTTCAAAGTCGATATAAACCAGCGGTTCACATGGAACAGAAACTGGACCACATGATGGAG
CAAGTATCGAACACGCACATGTAGATACATTAAAGCCCAGCCCTTGAGCACAATTCTGTGTGATTTGTATGCCGTCCACCATTTGGCCAAACACACATACGT
TGTTTGGGTCTTCGATTAAAGTGCAACCAACTGGTTGTGGTGCGATACAATTTTGGTCTGCCTCACAGGTCTGAGTAGTGTCATTGAACGAAAATCCATCGT
TACAGGCACGTTTTTGTCGAGTTTCGCCTTCACATTCGTAATATTCGTTACATTTTCCACTGACCTTGAACCGATATCCATCTGATTTACCTAAACACCGAGTA
TCAATACACTGCACTAGGTCTATATGGTTGCACGTGAGTGCATCTTGATCCCAGTAAGTACCAAACCCACATCGATGTAAGGTACCCACAGCGTCACCATTT
TGGTTATATTGACACTGAAGGTAAGTGTCACACTCCGGATTCTCTGGGACCACGTATCCAATACCATTCCTTAACACACAACCAAAACAAGGCCCAGGTTGA

TTAACTACAACCACAGTTTGACCCAGGGTGAGTGAGATGACAGAAGCCAAGGCCACAAACGAAACCAACAAAATATTTGCCATGATAACACTGTCTGAGG
TACAGATCTGACCGTTCCCCTAAACTCAACACCGACTGGTCAGATCTGTACCTCAGACAG
>Unigene36817_L1_2_1
GGGAGCCACACCTGTGTAAAAACTGGATCAAGACATTCCCACAGCAATTCAATAATACGGAGCCATTAAAAACCGATACGATTCGATTTATGGCTATATAA
ATTCAAATGATGTTTAGGACTGTGCACAATTCTATCGGCGCATATTACGGTGAAACAGTGTCTGTGCTAGAATTATGTATCGGATACTTGTTCTCTCATTGTG
CGTTACTGTCTGCTTTACGACATCTTTTAAAGGAAAAACTCTGCATCAACCGGGAGATGCTATAAATGTGATAGTAACCGGTGCAAGCCTCTTTCAAGATGG
AGGGAGAGAAAAGAGGGAAGCGGGGGAAATGCCATTAAAAATAGAGGTGGGAATCTCCTCGAGGTATGCAGTCTCCAAGCTTCGTCTCAAACGTAGTCC
ATTGGTAGATGCTTACGTTATCGACAACGGACAAAAGACCCAAGTGAACTCGATATTGGACACCCAGGCTGTGTATGAAGACCGAGAGAAAAGAGCTGCG
TTTATTATTAGACGTTCGGCAGCAAACGATTATCATTTGGAGGGAAGTCTTAATCATGGTGGACAAATGATTAATATGGCGCCAGCTAGTCGTACTAAACG
ACACACTTCTGGTGAAAATATGCATGAAGTTATCTCCTCCGATGCGTTTGATATTAACACCGATTTCGTTCATAGAGAGTTAAAAGACAGAGTAAAGAGGG
GTGTTACATTTTCAACTCATTATATCGAAATAACCTTCATTGCTGACTGTGAGATCTATGCCAAGTATTTAGCTGAATATGAAACAGACGTGGCGGCTACAC
AAGCTATGATCCTTCATTACACCTTTACAAGAACACAGATACAATGCAGATTTGAGTCTCTCAAAGCCAGGACCTTGTACTTCCGAATAAATATAGTTGTTAC
TGAGTTGATCATTATTATGGATCGCACACATTGTACCTTTACAGAGACGATTAAAACTACTAAAGGCCAGGTTGATGCGGACACATCTTTAACTAAATGTTC
TGATTGGCTTGGATATCCAGTCGGTAATTATACCTTCAGACCTACCGATCACTATATGTTCTTCACTGGGTACGACTTGACTCTGAATGGAGATGTACGTTGT
ATAGGTCAATCTTATTTCAAAGGTTTGTGTCAAGTAACAAAGCCGAATATCAAATCTGTATCCATCGTTGAGAATCACATATCAGCTTATATTACTGGACATG
CTGCTGCTCAGCAAATTGGTTATATCATTGGTGCAAAGTATGACGGAGAAGCTGGTGTAACTTGTACTAATGAAAATTACGTCATGACTCGCTATATGGAA
ATACCAACTGCAAACCAATTAGATGCTCAAAGAAGTTACGGATTTTCGTCCTGTTCCATTAACGCAATGAAAAGATTCTTGTTCGGAAGTCCTATATTGTGT
ACTCGAGAAAACAGATACAGCGGTGACATCGTTTTTGGGGTACAAATACCTGGTCAATTGCTTGATGCCGATGCACAGTGCCGTTTGGAATTTGGCGAATT
GTCTTATTTCTGTAGGGGATCCTACATGAATGAAATGAACTTCGATAAGATGTGTTATCAAGGGTATTGTTATAATCCCGTCATATACGCCTGCACTGCCATT
GTCTTCACTGAATTCACTACATGTGGTAACAGAAAGTGGTGTTTTAAAGGCAAGTGTATTAGCAACCCATCAGCACCAACAACCCCAGCTGTCGATTGTCCA
CAAGGAAACAAACACGGTGAAACGTGTAGTCCTCAAAGTTGTGGAACATATCCACTAAATCGTGTGATGGATTGCTGTGCAACATGTAAGGATACACCAC
GAATAAAACCAACAACCATATCAACCACATCAACCCCATTTTCACATAGTACATCCGAGGTTTCGTCGCCAAGTATATCCGACTTCACGTCGTTGAGTGCGT
CACCGTCTATTTCCCCTGCCGATAGTCCAACACCATCACCAACTACAAGTCCTACAACATCACCTACTACAAGTCCTACAACATCACCTACTACAAGTCCTAC
AACATCACCTACTACAAGTCCTACAACATCACCTACTACAAGTCCTACAACATCACC
>CL4170.Contig1_L1_2_1
TTAAGCAGTATTTTATTCAAGAGAAGATTAGTATATTGATTATTTTTAAAAACATAAGTGAATAGGAAGGATGCAGTAATTAACAAAAATGCTTACATAAAG
CTGTTTCTATAATTATGATCTTGTAACATATTATAGCAGATCATGACGCATGCTGGCATTACACAATGATTTCTCTGAGTTACATTCCTGATTGTTTTCAAATT
TCAGTGACCTCACTACAGACCCGGGACTGTACAGCGCACTGGACATTTCCGGCTAGCCCCCGGATCCATTCACCTATTTTGAATTGTTCGTTTGTTTGTGATT
TCTGCGTGTTGTATCTTGTCTGTAAATTTGTGTTTGTGTAAATTATGTGTGGTGCAGTCCCCCTGCACTTTCTAATTAATATATATATATATATATATCACGAC
TGAGAAAGATATGTATCTGTAAAATACTGTGTGATTTAAACTAAAGGTATGGATAAAATATGTAAATTCAAAGCTGGAAGCAGGGAAGACGAAAATGATT
ATTTCGATAACTGTAATATGATCATATCATTTCCTGGTCCCAACTTTTAATTAACCCATGTCCGTCTCAGTTTAAAGACACATTATTTGAGATTAGATAACAAG
TTGGCAGTTCTCACTATAATAGTCAAAAACCAAACGATGTAAAGGCAACATGCTGAAAACCACATTCAAATTATGAACCGAGAACTAAGCGATTGAAATTT
CCGCCCAGTCTGATCATCACCACTAAAGTCAGTACGACAAACAGCATAGTCTCGATTATCCATTTAATCGTTAAATCGTGAAGGAAAGCTATGCATAATCCA
CCAAAGATCGCAGGCCAGCGTTGCCATCCAGAGTTGATTATGGTCTGGATAAGTTAATGTATGTCTAATTAATAATTTAGATAACATTTCAGGCCTAAAGAA
AGACCTACAATAGACAGATTTAGCCCTTGCATGATGTATGTTTAATCTAGAATTGATTAATAAGAACACGTGCCCAGAAAACTTGTAGTCACTTGGCCTAGA
CAGTTGTCCCGGTAAGCATACGAGAAATTGTAGGTATTTCCATGCTCTCGCTTGAGTTGCTTAATGCATGCTACGTCATCTGTTAGGTTGTCATCTCTAAAAA
CGGAACAACGTTTATTGCAGCCATTTTTTGTCCTCCCATTGCCATTATCACAATTCCAGTAATCGTTGATCTGGAATAATCCATGGTCTCTGGAATTGTCAGA
ATTTGGAGGACTTTGAGCAGCCGTATTAAGTGATGATTCGTACTGAGCCATACATACCCAATGATGCACATCGCTTCTAGGCACGCCGACTCTCACCAACTC
CTTAGCTAACTCACACCTTGAAAATACTTTGCTCTCTACTGCAGCAAAAGTAAACAGGGCAACCAAGCAAACGTAAGCTAACATGTTGAAGGTAAAACTCTT
CGGTTTATTAGATAAGGTTTAGTAAACTGTGTAGGCCTATTTATACCTGCATTCGAGGATTTAAGATCGCAAAAACATAAAAACTGTTGTCATTTATTACAAT
AAACAGCCTGTGTTACGGTGACATATGAGATGTTGCCGGATTTAAATTGTATACAAGTTCATATTCTCTTTTTAAATTTGCTATTCTTAAGAATTCCTCTTGTG
CGTTGGGAAATAAACAAGAACTAATTTGACAGTTATGGAGTCGAGGACACGACACCACGACAATAATAACACGATTTTACGACAGCATTAATTGTCATGAT
GACGTGTTATATCGGGTTATG
>CL5700.Contig1_L1_2_1
CAATCAATGGGGAACAGTTTGTGATGATCATTGGGACAATAAAGACGCGTTGGTTGTTTGTAGAGAGCTTGGATTTCCCTCGTTAAGTGCTATTGCAAGGA
GTAGTGCACATTTTGGAAGAGGAACTGGAAATATTCATCTAGATAATGTTAACTGTACTGGAAGCGAATTATCAATACGATATTGTAACCATGCTGGATGG
AGCACTCATAACTGTGGACATAATGAAGATGCGTCTGTTACTTGTAGCCGTGTACGTCTAGTTAACGGCTTTGGACCATTCGAGGGTAGAGTTGAAATATA
TTACAATAATCGGTGGGGAACTGTTTGTGATGATAATTGGGACAACAAAGACGCGTCAGTGATTTGTCGAGAGGTTGGATTGTCTTCGACAGGCGCCATT
GCTAGAGGAAGTGCATATTTTGGACGAGGAACTGGAAATATTTTACTTGATGATGTTAACTGTGCTGGAAGCGAATCATCTATACGATATTGTAATCATGA
TGGATGGAACATACATAACTGTGGGCACGGTGAAGATGCATCTGTTACTTGTAGCCATGTACGATTAGTAGGAGGCTCAGGGCCATATGAAGGAAGAGT
GGAGGTATATTATAACCGACGATGGGGAACAGTTTGTGATGATAGTTGGGACAATAAAGATGCATCAGTTGTTTGTAGAGAGCTTGGATTTCCCTCGGGA
GGTGCCATTGCTAGAGGAAGTGCTAACTTTGGACAAGGAACTGGAAATATTCATCTTGATGATGTTAAATGTAGTGGAAGAGAATCATCTTTAAGATATTG
TAATCATACAGGATGGAATAGACATAACTGTGGACATTATGAAGATGCGTCAGTTATTTGTAGCATTTCTAGAATAGAAGGTGGTATTGGACCAAATGAAG
GGAGAGTTGAAGTATTTTATAACGGCGAGTGGGGAACAGTTTGTGACGACTACTGGGACAACAACGATGCCACAGTTCTTTGTAGAGAAGCTGGGTATCC
TTTGGGTGGTCGTATTGCTAACGGTGCTGCCGATTTCCCACGGGGAACAGGAAATATTCATCTAGATGATCTTAACTGTAATGGAAGAGAATCATCTATAA
GGTATTGCAATCACCGTGGATGGAACGTGCATAACTGTGTCCACACAGAAGATGCATTCGTTATATGTAACAATACATACGCAATAAGATTGGTAAACGGT
TCAGGACCACATGAAGGAAGAGTTGAAATGTATTACAACAGAAAGTGGGGAACAGTTTGTGATGATAGTTGGGGATCATTAGATGCATCGGTTGTTTGTA
GACAGCTTGGATTTTCATCGTTAGGTGCTATTGCTAGAGGAAATGCATATTTTGGGCAAGGAACAGGGAACATTTTACTTGATGATGTTAACTGTAATGGA
AGAGAATCAGCTATACGATATTGTAATCATGCTGGATTGAACACACATAACTGTGGACATTATGAAGATGCGTCTGTTACTTGCCAGGTTGCATATTCAGTA
CGATTAGTCGGAGGCTCTGGTCCACATGAAGGAAGAGTTGAAGTATTTTACGACAACGCGTGGGGAACAGTGTGTGATGATAGTTGGGACTACAAAGAT
GCTTCGGTCGTTTGTAGAGAAGTTGGATTTCCGGGAAGTTCTGTGGCTAGACCTTCTGCTTATTTTGGACGGGGAAATGGAAATATTTTACTTGATTATGTT
GACTGTAATGGACGAGAATCATCTTTAAAAGCTTGTAAGCACAATGGATGGGGCAAGCATAACTGTAATCCTAATGAAGACGCATCAGTAACATGCAGCTT
TTCATACTCAATAAGATTAGTAAATGGTTCAGGACCACATGAAGGAAGACTTGAAGTACTTTATAATAATCGTTGGGGAACAGTTTGTGATGATGGTTGGG
ACTCCGCTGATGCGTCAGTGGTCTGTAGAGAGCTTGGATTTTCTTCGAGAGGTGCTATTGGAAGACAATATGCACATTTTGGACAAGGCGCTGGGAGTATC
ATTCTTGATGACGTTAACTGTAATGGAAGTGAGTCATCTTTAAAGTCTTGTAATCACCGCGAATGGACCGAGCATAACTGTGGACACAGAGAAGATGCATC
AGTTACCTGTGGATTTACATACGCAATACGATTAGTTGGAGGTTCGGGACCACATGAAGGAAGGGTTGAAGTATATTATCATAATCGGTGGGGAACAGTT
TGTAGTAATAGTTGGAGCTCCAATGATGCGTCAGTTGTTTGTAGAGAACTTGGATTTTCTTCGGTAGGTGCCATTGCCAGAACCGGTGCTTATTTTGGACAA
GGAACTGGGAGCATTTTACTCGATTACGTTAGATGTACTGGGAGAGAATCATCTTTGAAATATTGTAATCATAATGGATGGATCAGGAATAGCTGTGGACA
TAGTACTGACGCATCGGTTACTTGTCGACGACGCGTTCACCAAGTGGACCCGATTGTGAACGTGACGTTAGAAACCGTGGAAGAAAGTAGAAGGAAGATG
TACAGATTTTACTGTGACTTTACGGGACGAAGAGATGATTTGATTTATAGCGCTGCATGGTTTGTCAATAATTACCTGGTATATACTAGCTCTGCAGTTGGT

GGTGGCACGAACCTGACTAGTTTCAGACATAGCCTTAGTTTAACAGACGATCAACTGAATAGAACAGTGGGCTACGATATCCGCTGTCAGGTACTAGCCTC
AGATAGTTTAACAACAACTAGATCCAACAGTTCTTCACGTTTCATAGGCATTAAGATATTGACACCAGTAATTCGAGTCAGGGAAGGAGAACATGGTGTAA
TAAGAATAAAAGCGGATGCTGAAGTAGGATGCTATAATATTGCGCTGGGTTGTAGATATGTCGTATCAACATTAATACCGGATAGTGGACCTTGTGATGC
GACGGCTACAGTTAACAACTATTGTGGTGTCAACATTCTAGAAAATATTGCCAGTGGATGGGATCAAGAATATGAAATCAATATAACAGCTGGTATCAGTA
ACCAATATGGACAATCATCAAGAGAGTTTGAACTAACTTTGGGGAATTTTGGATCACACTATGACCATTGGACATGGGCGAAATATACCATTTTCCCCCGCG
TTCAGGTTATTGTTGAGAAAGATACAACCAGTACTCAGTCATTGGTATGTTCAGCAACTAGTGATCCGAGGCTTGTGACTTTTAAAGGCCTGAGAATTAACA
TTCAGAGTATTGGAACATTTTCACTGTATAAAGACAGAGAACATGATATTGAGGTTCAAATTCAAACCATTCTATGTAGCGGTCTTATTGGACATTGTAATT
GTGGAATGGTGGCAAGGATTGGCGGAACAGCTTATATGATAAGTGCTTGTAACTCCAACCACTGGATAATAGAATATGTTTTGTGTGATACTGCTGTAGAT
ATTTTGAGAGTACAGCATACACAATCGAATATTTATGAGCTTACTTTGCCTACTGGTGCAACAGTGAGAGCTGAATTAATAAACGCAAATCCCAAATATGTT
GACGTCTCGGTCACTCCTTCTGTATTAGATACAAATAGGTCACGTGGTTTATGTGGCCGAATATCTTCAAACATAACTGAACAACTTGTTCTTAGAAATGGC
AGTACAAGCTCCGATAGAATAAACTACAATCCGAATAGAGGATTTCAATCAGCTTCAGAATTTGTCAACTCGTGGGGGGTTCAAAGAAATCAAAATTTGTT
CGACCAAGCGGTTCTGGATGTCCTGGTACCGAATACAGCAGTAACTATAACATGTCGTTGTAATAAAGCAACGTCTAATGATGTTTCAAGTACCTTGTCATG
TGATGCAGACAGTCCAGTTAAAATATGTAAAAGTAACCCAAATTATCACCCCTTTTCAAAAAGAACGTGTTCCAGTCGCTCACCTTCCCGTAGAAGACGAAG
ATCTGTTCCAGCTGTCCTTCACATTGATGAGGCACCTAGGCGAATCAAAAGGCAAGCAGTCTGGACGGGAAATTGGACAGAAGAAACGGCGACTGTGTTT
TGCACCAGGTTATTAACTGCCACTCCTATAATTGAATTTTGCAAGAGGGAAGTTAAAACTGTGGATATAGACCTTGCTCTAGAAACCTGTATAGCAGACATT
AAACTTACCGGTACAACAATCTTCGCAAAGTCTCCAGTCTCCAGTGCACAGCTCCAATGCGTAGGAGAAGCGTACTTGGACCCCATGTTACAAGTGGAAGA
AGCAAATAAAACATCAGTCCTGAGACAAGTTGTAACACTGGACTGTCCGAACAATTGTTCAGAACAAGGAACCTGTAATAATGGAACGTGTGTGTGTTACA
GTAACTATATCGGTAGCGGGTGTGAATTTGATCTTCGTGATGAGCCATTGGCTGAAGAATTGGATTTTGAAGGGCTTTGTGATATAAGAACTGAAGAATGT
GATGATATTGGTGTTATTGGCGGTTCTTTTATACAAACAACAGACTCAACATGTCGAATTACCCCTTACCAACTAGCAGCAAATGGAACTACAACCTCTACTT
TGGAACCAGTTGTCAACGATGCATCGATCGAAAGCGTGGGGAAAGTTATTTGTGAATTACCATATTATACTAGTTATAGACAGAGGAGATCTGCTGGACAT
ATTCCTCAGACACCATCCATCCAAGACACGTTTACAGCTTATACTATTTCAGTATCCAATGATGGTCAACATTACAGCAATGCTCTATCAGTTGTGTATTTTG
ACTCCAGATGTATGGACTGTGACGTTGACGGGGTTGTCATTAATTGTCAGTTTTCGCAAAACCACTGCGTGTACCAAAACCGATGCTACAACAATTCGGAA
AATTATCCCTTGAATGAACAATATATCTGTAGAGCTGATAGTAATGGCAATAGATGGGAGATTGATCCCAGTAAAGCTACCGACCCATGTTACATTTATCAG
CCATTTACCAATTCTTCTGCAAGACTAACCAGTTACCAGTTGGACCAAACGGTTATAAACGACCGCTATCTATCAGAGGGTTGGTATGGAGAACCTGGAAA
AGTCATTGCAAATGTCACAGCTCCTCCACAAGGCCGTTGCGGAACTGTATACCCTATATATCTTCAGACTGCACCCACTAAAGTGTTGAATGTGAATCAATC
TATCACAGCTTGTGTAAGAAGATATTCTGCTCTATGTATAGAGTCCATTGCCATCAAGGTCAAATACTGTAGAAACGATTTCTTTGTATATAAGTTGAATAGT
TCACCTTTGGAGTCATCTGGATATTGTCTAGAGTAACCGACAAAGGCAACGTTACCTACTACAAGAACAAACAAGAGAATGCATGCTATGAACTGATGATC
CCTTTTATATTTTCATTTTTCATTTTAAGTTCATATTATAGCCAATTATAGCAAAATTCTATTTTGTGTATGATGCTTGTCTGCTAGATATTTTTACAATGTGGTT
TATAGGAATTTTTAGTCTAACAAAAATGTCTAAGATATATTAAGATAACGCTTTCTTTGTATCCTTAATTACGCATAATAATGTGTAGGTTATAATATAATTTG
GCATGGGTTTATTAATTCCTTATAAAAATATTTTCTAGTATTTGTTATTTTGATCTTTAAATTTCTTATTAACTGATTGATTATTATTTGCTATTTTCCATTACAA
AAAGGTGTCGTAGTATTTGCTATATGTTATTTTCCACTTCATATTACTTATAAAATTAGTAGATTAGTTAGTAGATGTTATTTTTAAGTTAACATTACTAGTAC
ATGTTTTTATTATTTTTGCATTCATTCCCTGTGAACTAGCTTTCTTTTAAAATGCATTTTTAGGGGTTTAGATGTTGTTGGGTTTTTTTTGTTTTTTTTTTCTTGG
GGGGGGGGG
>Unigene11944_L1_2_1
AAAGCTTTTGCAGTTTTAGTTCTTGCTGTTGTGGTTTACAACCATGCAGCTGAAGGCTTTGATTGTAACCAATGGACGAAATTCTATAGTCGAGATCAACCA
TCTTGGACAGCAGACTATGAAGCTCTTGCTGAGATTCGAAAGGAAAACCCCAACGATCACATCTGTGCAGTACCATCAGACCTTGATGTCCAAACACTGAA
CGGAATTGGTTATAGAGAAGCTAGAGAAGTTGTAAAGATTGGTCCCACTTTTGGTTTCGCATGCTACAATAATCAGCAACCTGACGGCCACTGTTTAGATT
ACAAAGTTCGATTCTGTTGTATGCAACAGACACCAACATGTAAAAAATGGACCCAGTTTTACAACAGAGATGCTCCATCTGCTTCTGGTGATTGGGAAACTT
TGAATGATCTACGTAAAGCCCACCCATCCCTCTGTGCAAACCCTTCGGCCATTGATGTCAGATTGGTTAAAAACGGAAAGGATTTTAGAACGGCTAATGAG
GTTGTAAGTGTGTATCCATCCAGAGGTTTTTGGTGCCAGAATAAGCTGCAGAAAGATAAATACTGCGAAAATTACAAGGTCCGATTCTGTTGTCCATAAAT
TCTTCCATCTGTTATTCTCTTATTAAATATGTAACACTAATTGTTAGTGAGATCCGAAAACATGTAAAACAAATCTTTATTTCTCAAAAAGAAACTTCTACTAT
CATTTGTAAAAGTCCCTTTTGACAAGTACGGCCAGTAGATGATGTAACACTTATTATTAGTGATATCAATATGTAAAACAAATCTTTTTATCTTCGAAATAAA
CCTCTGAT
>CL1231.Contig2_L1_2_1
TTTTATTTCTTTAAATTAATGTTGGTATATAAATTTAGCATTTCTTCCACTCTCTAACACTTTACTAAAACACCATTTTCATAGAAGTCTGCTATACACCCACCA
CAGTAGTTAGAGACGCATGTGGCATTTGGATGAGATGGACATCTTGCAAACCTACATGGATCTATGAAACAGTTCACCAACTGCACGCCTTTAGGACATTG
TCTACACCGACATGTTGGACAATTATTCCTATCTAATTCTTGTCCATGCTGACACTTTATCAAACCAGCACAAGGTAGTCTAGGGCATACTGGTTTAGGTGTA
GGTTTGAAACCACCTGTTGGGTGTTGACATCTATAACCACAACCTTGTCGACAACATTTCTGTGTAAAAGGACAATCTCTGTCGTTACTGCATTCGTCGAATT
TATAACATGGAAACTTGGCAGCAACAAATTCCGGGCAGAAACCTTCCTTTACGATTGACAATTCGGATTTTCTGGGATATTTACAAACTTTGCCACATCCATT
CCTACAACAAATCTGACCGTCGTCACACTCACGATCGCTTAAGCAGTTGAACCTAGGATCAAAGAGACAGATCGTTGTTAAACCACTTGGTTTTGGACAAC
GTGGGTTATCCTGAGATCTCACACATACAAAAAACAACGCAAATAGTAAATACTTCATTATGTCACAAACATGCAGTTTAAAGTTCACACAAAACATGGACT
ATACAACTTACTGTTATATATATACAACTGGTTAGGACCAATCCGATTGCTAGTTTCAGTGTATCCGCTTTAAATGACAATGGGCTTGACATTGCTATTGACA
CCAACATTTTGGCTAATTCATTCCGACGTAATTTCTGTCATTTTTGTAATTTTTGACATTTAGGTGCGGCTGTAATAATGTTAGGGACTTAAGGTAAATAACA
AAACATAATGTATCCGCTTAAAAAT
>CL5339.Contig3_L1_2_1
ATGGATTTAAGGCGGCGGAGTTTTAACTGGCAACATAAATCACAGATCATCGCTCAGTATATAATGTATTATAAAATGACAACTACAGATACATTGTTGATT
TTTCTTGTTTTATCAAGTCAAGTTTGCTCTATATTCGGTGCCATCAATGTTGCTATTAATAAACCAACACGACAGAGTACGATTAATCTTGGTGTCAATACTTC
AAGTTGGGCTGGCTTAACAGCGTGGTGGTGTATAGATTTACTACAGGAGTTTCATATCAACACCGTTAGAATATTGGAGAGAGCTCATGGTGGAAGACAT
GAACTGAGTGATGGATTTGAGATGAGAATAAGTTCTAGTGGTCAGTGTGATAAAGCAGGGTTTACAGCAGCTACACCATGTTTTAAAGATAGCGCATCAA
CAACAGATTCAGTACATACTATCGAGAGATGCAATCATCCAACAATACCATCATTATCCGCTAGATATATTTTTATATCTGTTAAACGCACTGATGCAAGATT
GGATATCTGTGAGGTTCAAGTATTTGCAGATGACTGTCCGGATGGTTTCTATGGTAGCAACTGTAACATACGATGTCCCACATCCTGCCTGAACAACACATG
TGATAAGATAGGAGGATCGTGTTCTCACGGTTGTTATGGTGATCACTATGGTAAAACATGCCAGTCTACTTGTAGTAGCCATTGTACTGATGGTAAATGTG
ATGACATGACAGGGCGATGTTTTGGATGTAAAGATGGTTACTATGGTAAAGAATGCCAGTCTACTTGTAGTAGCCATTGTACTGATGGTAAATGTGATGAC
ATGACAGGGCGATGTTTTGGATGTAAAGATGGTTACTATGGTAAAGAATGCCAGTCTACTTGTAGTAGCCATTGTACTGATGGTAAATGTGATGACATGAC
AGGGCGATGTTTTGGATGTAAAGATGGTTACTATGGTAAAGAATGCCAGTCTACTTGTAGTAGCCATTGTAAGGATGGTAAATGTGATGACAGGACAGGA
CGATGTTTTGGATGTGAAGCTGGTTACTATGTTGATGTGTGTGCAGAATCGTGTTCTACGTGTAATGGTCTATGTCGACAATTAGATGGCGTTTGTTTGACT
GGTTGTAAAGATGGTTATTGGGGATGCAATGGGTTGTGTCTACAGACATGTTCCTACTGCTCTACAGGAGGCTGTAGGATAGAAGATGGCGTATGTTTTAA
CGGATGTAAAGATGGTCTGAACAACACTAAATGTAACCAGGATGCAGTATGCGGATCACTACCTCCCAGACTACATGCAGTTGCAGATCGCGTGCAAGAA
CTATATCCTGCTGGTGCTATAGTTAACTATAAATGTATTAAGGGGGCTTATCTACAGGGGTCACCACAGGCTCATTGTCTACCATCTGGTGAATGGGATATT
CAATTATTTACATGTAAAATTGCCAGGACATGTGATGAAGCTCATCAACTGGGAGTACCAGTTACACCTACTCTAGTCGTTAGACCGGATATCAAACAACCA

GCTGTAACTGTATCTTGTGAAGTTACAGACTATGGTGTTTATACAGTTATAGGTCATGGATCTAACAGAACATATGTACATGGCTATGAAGCATCTAGAAGT
TATAATGGCACTATTAACTACAACATTGATCTTCATCAAGTTATAAACATAGCTGATGCATCGGCTGAATGTGAACAATTCATTAAATTTGAATGTTACCATG
TTCACATAGTCAGTGATGTTGGCTTAACCACACGAACTGGCGAACTAGCTACTTATTTAATTGGAGGTGTCAAGGGTCAAATAGACTGCGCATGTCATATC
AGTAATATGTGCGCTAATGATTTGAAGTGCAACTGTGAGAACAATGATGAGCAATTGCGAGTTGACGAGGGGTATATTCGATATAAGGAAGATTTGCCGA
TAACCGCCATTTTAGTAGGCGATACGGGGCACAGTCAAGAGTACGCATATTATACCGTGGGAAATCTGAGGTGCAAGGGTTAGATAAGCAAAGATGAAG
ACCTGTGTTTATTGGTTAGACGCTGTAATCTGAAGATTTCCTAATTACATTGTAATTGTAATTCAGTCGATTAAATGAAATGTATATAAATCTTTATTAATTGT
AATTCAATAGATTAAATGAAATGTATATAAACTCTATAATCATGCTATCTGTAAATGATGAGCAATAAGAAAAATATTTTTGTGTATAATAAATTAATTAATG
AGAGTATATCTCAGATACTAGGCCTGTCTTTCTAACCCCGTCGAGTTTCTGCCTTTCTAACCTTACTCAATGACCTACCGCCTCCCCCCGAGATATATTAGTCC
GGTGGCTGAAGGGGGGGGGGGGGGTCGCCACCCGAGCTGTGAATTTATGAGGCCAAGTTTTTCTAGACCAGTGTTTTGCGTAGATTCCGAATATGGGAGG
TGATCAGGCGTTTGAGGGGGGCGTTTTCGAGATATGGGGGGTCCAATGTCTACAATTGGGGGGCAATGTCATTATGTTGACATAAATGATTATCATGTTATA
TATTAATAAAACATAAATTTCTAGGCCTATATTTCAAGTTTCGTCAAAATTGGATAAAAATTTTGACCTGTCAAGTGTCCAAAAACAGATTGTGAATGGACG
GGGCGAT
>Unigene15438_L1_2_1
GTCACTGAGATTTTATTCCACGGAGGGTACAACTTAATAAGCGGACTCTGACTGGAATGGTGAGCTATTTCTCTCTGTATTGATATTTTGGGAATTGGTGAT
GAGATGACTCAGGGCTACATGGATTATGATCACTACCCTAAATTAACCAAGCATCAAAAATATCGAATCTATGAAACAGTCATCAATGTACAGTTCCAGGTT
GTGTAAGATCAAGACAGCTAAGATGTTTGATCAGACCGTGAAGATAGGCCTGCCGTTGCTTTGTATAGTTGGACTGGTGCTGTTCATCATTTTAAAGCTTTC
ACCAACCAGTATTTATAGTGTGGATAGCATCAAGAAGATTATCAAGATTAACCATGCGAAACCAGGACCTAGAGATAGAGATCAGATAAAAAGACATCAA
TATGTTGATGCTGTATGTAAGAATAATACCCTGGGAAGTACTGGTAATATCCTTGTTTCTTCAGCCAAGAAAATGTCTTATTGTATTGTACCTACAGCAGAG
TCGGAATTCTGGATCCGAACATTTCGGTACCTCAACGATGAATCCAAGGAGGAAATAGACACAAGAGATTTGACGAAACTAAACAGTTTGCTTTCTCAGAA
CCTGCTACGACAGAATTTAAAATTATACCACATGGAGAAAGAGTCAGATGAAATTGTCGTTGATGCAACAAACAAGATTTTGTTTACAAGAAACCCCTACA
CAAGACTATGGATTGCTTACATAGAACATATGTTTTTACCAGACTTATGGAACGAGGTCGGGCGTAAAATTGTGTCATTTCGAAAATCTCCCAAACAGGAA
AGTCTTCTGTGCGGAAATGACGTAACATTTGAAGAATTTTTGGATCACGTGCTACGCGAGGCGAAAAGCCCAGCAAATATGAAGAAAATTTGGAGGCCGG
TTCAATACCTGTGCAATCCCTGCAAGTATAAACCAGATTTTATAGGAAAAGTTGAATATTTTGATGTTGATGCAAAGCATATACTTCAAAAGTTCGATTTATC
TATATTACTAAAATCATATGAAAGCGAGAACTATGAAATTGGTGAAATGAAGAACATAATCCGTCATTATTTTCACTTGTATAACCAGGGTACCATTAGCAA
CTGTATTTCTAGAGCGGGATTGGCTCTGAGATTGTGGACTAGCTTTCAAGTGCGAGGTTATCTCCCTTTACAATCAGAATCGGCTCTACAGCATATAACAGA
GAGAACTTTTGACGTCAATATTTTCAGAGAAATCGTGGTCAATATTTATGATCGTTACAGAAGTTCTAGATACCTGTGGAAGAACCAACATAACAAATTAAC
TACCCACGCTTTCCAGGAAATACCCTTCCGAATTTTTAAAGGTCTAACAACTGTCTATGAGTTTGACTTTAAAGCTTTCGGATATGAAATGTTCCCAAAACAA
TTTTTAAAAATACCCGGGGTTCCACGGATCTAAATAAAAGGCAGATAATTACGTATTTCCCTTGGTTTTAATTACTTTAGAATTTCTTTGTAAAACTGAAGAC
TTTAATTTGTAGCAATGGAGTTTTCACCACAAACAAAGGATGGCCCATATCATTTCAAGAATAAGATAAATCCAAGCTACTGAACTACTTGAAAACTAAAAT
AATGTGTACGAATGTGTTATGACTTTTTAAAACGCCTATATCTCTGTGATGGATTAGTCTGCAATCCAGGTAGAACCACTCGATAATTACAACATTCCATTGA
CTCAATGCCAAATCAATGCCGCTGTTAGAGAATACTGAACGATGCACAGCAATCCACACAGGGACAACTGCTAGTGTTACTGTTATGTTTTTTAAGGATTTG
TTTTATACAACCAGAAGATGCATGAAGATTTTTATTAAATTAAGCAACTAATTTTAATATACATAGCTGGTTGAAATGAATTTTAATTTAAGCTGACATTAAA
TGCAACTAATAAATACTAAATTCAAAATAAATATAATGCACAGTCCGTGGGCGGTCGAATATCGTAAGGTTTTTTCGTTCATTAATCAGGGCTTGTATTTCC
ATCACAGAAATAAATTATTTAGTTTTTTGTAGGAACAATTTTTGCTTTAAAGTACCACATTCTACATTGACCTTCTGGTGTAAATGTGTATTCATTTCCAACAT
TGACACTTTTAAAGTTCCATAAATAGTTGTTCAATCTTAATGCTCCGGGCCCTGCGCAGTTCCAATCCAGTGGCACACCTACGCCATCTTTAGGGTATAACCC
CAAGACGTCGCCGGCCTGAACCTTTATTCTATCTTCTGGTTTAACTGGTACATACTGTTTTCTCTTAAACCTGGTTGTCACCAGATTTTTTCCCACTAAAGTCA
TACTTAATGGGGAACTGGTTGGTCTAAACACAAAAAACCCAACTTGTCCTGACGTACGGCTATAGAACTGCCACTCAACTATTTCACCAGATATAGGAATTC
TAAGAGAATCACTCATTCCTGTGTGTGCCCAATTTCTCCACCCTACCGCCTGGCCATCCCTTAAATCCACATCGTTACTAAGAGATGGTACCTCATTCAACGA
CTGACATTGTAGAAGTTGACCGGATAATTCTCCTGATCTCATACAGATTCTACTTTTACTGCCTGACTTGTATACATAACCAGTATCACACAAATAGCTGACG
TATTCGCCAATTTTTAATGACGTCGTTGTTCCGTTCTGTTGAACTCCTTCACCACCAGATTCAACCGTGCATTTTTTATCTGATACCTCACATATATTACAAGA
GACTGGACATTCACTCCTAGCCCATCCACCATTTTGACAGGATTGGGGTAAGGCAGCCATAATATCTTCACAGTTTTCAATCTATCTTTACATAGCACGTGC
TTTTTGGTGGCCCTTTTACATACAAAATCTTCCGGCCAATAGCCATTGGTTGCTACCCATTTTCTTCCCTTATCTTGACCAAGTAATGATGCGTATTTAAAGTC
GTATCTGTCATACGGCGGAGCTTGCCATGGTAACCATTCAGTAACATTTCCATCTGGCCATACCCACTTGCCATCCTTCTTCTCCAACCCTATCATTACTTTAT
GCATAGTTTCCCTGATGGAGATCACAAAATCTTCTTCGGCTTGATCTACAACTGTTGTTAACGTAGCTCCATGTTCTTTACAGATCTGTTCAGCTGCCACACG
TTTCACTGGTATACCGAAATATTTATAACAGCCCTTCTTATATTCTGCCCATTCATATGGACATCCACCACAGACATAACCTACAGGAGACCAAGTTCCATCT
GTTAAACAGAAACTCCTGTATTCTAAATCTTCAGTTTTACATTTATATGTTATAACATCTGCTTCTCTATATTCATCTTTCTTTCCAGACTTTATCTCAGCATCTT
CTAATATTACCGGTGCCGGACATGATACATCTAAATATGATTTTTCCTTTTCTGCAGATCCACCACCCCCGCTACATGCCAAGGCACACCCTGACAGGGCAG
TATCTCTCTGACAAAAATCGGGATCTTCTTTTATCACGGCATCACAGTCGTCATAACTGTTGTGCATCTTCCCTATTTTACTGACGTCACAGTAATTTCTACTG
CAATCAGATGTTGCTGTATAACACCAAGGCATAATACCATCATTGGGATTACGACAATAATTCTCAATTAAATCATCTTCAAAATCACCGGGTCTCAGCGGG
TGATGTTCACAATGGGTAACTTGTGACCAGGGTAAACATGGTTCAAACGCATGGGTAATATTGACCTTGCCTCTGTACGTTCTGCCTTTATTTAATGGATCA
TAAACATAGTCTTCATCTTGTATAAGTTTAACCGTTGCCTTAAATCCTTTCTTAGAGACAGCACTATTACTTCTAAAGGTCAGACTTATATTATTATATACACT
TACAATAGTCGTTCCTAATTGACTACCACAATATTTTATTCCAGCTTGTCCCATTCTGTTTTGTCGTATTTCTATATAATCATGACAGGTTGATGTCCCTTCAAC
TTCAAATTCATCAAAATCAATTTTAATGGTCAGATGTTCTGGTCCCTTGATGAGCCAGTTACATTCTGTTTTATCAGGATAATTTTCTGGATATCCAGGACTA
GTTATAGTCACGGATGGGTTACTATGATCTAGGTCAATGATTTTACCACATCCTATATTTAACGGATAGTAATAACAAACTGCTAATCTGATAGTCCCATAGT
TGGGATTAAATGTAACTTTAGGATGCTCTACATTTGTCGGAAAGCCAAAACCATCAACTGTGGCTGCAGGTTTATTCTGAACAACAAAGAACTCTTCTTGGT
GGCCCATACCTTCAATAATTTGGCAAGTTCCACTATGCGACACAAGAGCGAACGGAACAGTATTTGGTAACCTTACTGCAAGATCCTGAAAACCATCACTG
CGACAACAGAAGAAAAGTCTCGTCTGCTGCTCAAATACTCCGTCTGGTACAGCGCCAGTGGTTTCCGGTGCAGTTGTCTCCCCATATTGTATAAATGATTCG
GTGAAGCCTGCTGGACATTTTCCACCTTTTCTAAGAATACAATACACACCAGGTTCCCACGGTACCTCACTTTTGTTGTCTTCATCACTGGTACAAACCTGGA
TTTCAGTCTTTGTGCCGTCATAACCAGAACCGAGATGCCATAAATTGGAGTTATATGATTTGGTCGAGTGTACAACAACCTTGCCTTCTTTAAGTCCTTCTGG
GCAACCGTTTTCTGGTCTTAAGATCGAATAGGTACCACCTGGCCACTGCTTCATTACAGCCTTTTTCTTCACATCAGCACAGCCCGACGCAGCTTCTAAACCA
GTAGGACATGCACAAACACATTCATCTCTTACTAGAGTTAAATACCCTTCATTAACACACTGAAAATCTGCACTTGCACAATAAGTCTCTCCACATTTGTGAC
CTAGTGATACTTCCTTATATGCGTAAAAGTCCGTATTTTTCACCCTTAAATAAGGTCCAGAGTACAACTGTGACATAACTGGTTTACCATTAACACTGAAAGT
ATTCATATTGTAGTGCATGTCAGATGTGATATCATAACCAACCATAGAACTTAGTGGAGAAGACATTTGGTCTACGAAAGCCCTTTTCTGTGCCGGTTTAAT
ATTCTCCCAGTTTACCCGAATCCATCCTTTTCGTAAGGCCGACTGGTGTTCATGAACTAAACCTAATGTGTGACCTAATTCATGTAGACACAAAGGCTTTCCA
CAACAGTTAACAGTTTGATGCCTTCTTGAGTTACCTTGAATTGACCAACAACCGCCTTTTAGAACGTGTTCCATAACGCCATCATGAGTAAGCCCGTATTTGG
ATTTGGTATCTTTGTCATTTTGAACAAAACGTATACATGTTTGGGTTTGATATTTCAACATACATGCTCTACGTTCAAACTGTGACTGTGGGGTATTAAAAAC
TGCATCGAACTCAAATGGTACTATACCACGATCCCATCTGTTATATTCAGACGGCAATATAAACCTTTTCCTTCTGCCAGCGGATTTAGGTACTGGTTTGGCA
TCCTTTACAGTAACAAACGAGTTCTTTACATACGCCGTAGATGGGAATAGAGAGGAAACTGGGGGTCTTGTATTTACATCTCTTTTACGTCGTCGTCGTCCT
TCTTCAGTTTCATTCTCTCCATTAACTTCTTGATCTTGCTCTTGTTCCGTTTGTTCATTATGTTGTTCTTGTTCTTCTTGGTTTTCTTCTTGTTCTTGTGAATTAAC
ACCTGCATCATTTTGTTCCGCGCCATCATCTTCATTAAGTGAATCCAATATATCATTAGCTACATCATTCTCTCCATTAACTTCTTCTTGTTCTTGATCTTCTTGT

CCTTCTTCTTGACCTTCTTGTTCTTCTTCGTCTGTTTGCGAATTAACACTTACATCATTTTGTTCCGCGCCATCATCTTCATTAAGTGAATCCAATATATCATTAG
CTACATCATTCTCTCCATTAACTTCTTCTTGTTCTTGATCTTCTTGTTCTTCATCTTGTTCTTCTTCTTCTGTTTGCGAATTAACACTTATATCATCTTGTTCCTCG
CCACCATCTTCATTAAGTGAATCCAATATATCATTAGCTACATCATTCTCTCCATTAACTTCTTCTTGTTCTTGATCTTCTTGTTCTTCGCCTTGTTCTTCTTCTTC
TGTTTGCGAATTAACACTTATATCATCTTGTTCCTCGCCACCATCTTCATTAAGTGAATCCAATAAATCATTAGCTACTTCATTCTCTCCATTACCTTCTTCTTGT
TCTTGCCCCGTTTGTTCATCATCTTCGCCTTGTTCTTCTTCTTCTGTTTGCGAATTAACACTTACATCATCTTGTTCCTCGCCACCATCTTCATTAAGTGAATCCA
ATAAATCATTAGCTACTTCATTCTCTCCATTACCTTCTTCTTGTTCTTGCCCCGTTTGTTCATCATCTTCGCCTTGTTCTTCTTCTTCTGTTTGCGAATTAACACTT
ACATCATTTTGTTCCGCGCCATCATCTTCATTAAGTGAATCCAATATATCATTAGCTACATCATTCTCTCCATTAACTTCTTCTTGTTCTTGATCTTCTTGTTCTT
CTTCTTGACCTTCTTGTTCTTCTTCGTCTGTTTGCGAATTAACACTTACATCATCTTGTTCCTCGCCACCATCTTCATTAAGTGAATCCAATAAATCATTAGCTA
CTTCATTCTCTCCATTACCTTCTTCTTGTTCTTGCCCCGTTTGTTCATCATCTTCGCCTTGTTCTTCTTCTTC
